# Lactate promotes metastasis of normoxic colorectal cancer stem cells through PGC-1α-mediated oxidative phosphorylation

**DOI:** 10.1038/s41419-022-05111-1

**Published:** 2022-07-27

**Authors:** Shuang Liu, Hui Zhao, Yibing Hu, Chang Yan, Yulong Mi, Xiaolan Li, Deding Tao, Jichao Qin

**Affiliations:** 1 Molecular Medicine Center, Tongji Hospital, Tongji Medical College, Huazhong University of Science and Technology, Wuhan, China; 2 Department of Surgery, Tongji Hospital, Tongji Medical College, Huazhong University of Science and Technology, Wuhan, China; 3 Department of Breast Surgery, Peking University Shenzhen Hospital, Shenzhen, China; 4 Department of Gastrointestinal Surgery, Peking University Shenzhen Hospital, Shenzhen, China

**Keywords:** Cancer metabolism, Cancer stem cells

## Abstract

Uneven oxygen supply in solid tumors leads to hypoxic and normoxic regions. Hypoxic cells exhibit increased secretion of lactate, which creates an acidic tumor microenvironment (TME). This acidic TME is positively associated with tumor metastasis. Despite the increased metastatic capacity of hypoxic cells, they are located relatively further away from the blood vessels and have limited access to the circulatory system. Studies have shown that cancer stem cells (CSCs) are enriched for tumor metastasis-initiating cells and generally undergo aerobic respiration, which could be enhanced by lactate. We therefore hypothesized that TME-derived lactate may promote the metastasis of normoxic CSCs. In the present study, the abundance of hypoxic and normoxic CSCs was analyzed in primary CRC tumors. It was found that the proportion of normoxic CSCs was positively associated with tumor stage. Using two human CRC cell lines, LoVo and SW480, and a patient-derived xenograft (XhCRC), it was found that treatment with lactate promoted normoxic CSC metastasis. Metabolism analysis indicated that, upon treatment with lactate, oxidative phosphorylation (OXPHOS) activity in normoxic CSCs was enhanced, whereas hypoxic CSCs were rarely altered. At the molecular level, the expression of peroxisome proliferator-activated receptor-γ coactivator-1α (PGC-1α), a master regulator of lactate oxidation, was found to be elevated in normoxic CSCs. Furthermore, PGC-1α knockdown markedly reduced the metastatic potential of normoxic CSCs. Notably, both the PGC-1α-mediated OXPHOS activity and metastatic potential were impaired when hypoxia-inducible factor-1α (HIF-1α) was activated in normoxic CSCs. Together, these findings provide a therapeutic strategy against tumor metastasis through the targeting of PGC-1α and, thus, the suppression of lactate-feeding OXPHOS in normoxic CSCs may improve the therapeutic benefit of patients with cancer, particularly CRC.

## Introduction

The rapid proliferation of tumor cells results in high oxygen consumption, and the aberrant function of tumor vasculature results in inadequate oxygen supply, which in turn leads to intratumoral hypoxia in solid tumors [1]. Tumor cells in hypoxic regions (hypoxic tumor cells) exhibit an increased anaerobic metabolism of glucose due to a lack of oxygen supply, thus producing abundant lactate [2, 3]. The Warburg effect is a unique metabolic phenomenon during which tumor cells mostly rely on glycolysis for metabolism and secrete lactate, even with an adequate supply of oxygen; this suggests that both mechanisms (i.e. tumor cells mostly undergo glycolysis in the presence or absence of oxygen) may cooperate to induce an acidic tumor microenvironment (TME) [3]. Studies have shown that the high levels of lactate in the TME are positively associated with distant metastasis and a poor prognosis in a variety of cancer types, including colorectal cancer (CRC) [4, 5]. However, recent studies have reported that normoxic tumor cells located in regions with enriched vascularization prefer to undergo oxidative phosphorylation (OXPHOS) by consuming lactate in the TME [6]. Of note, it has been shown that metabolic commensalism may relieve the fuel shortage occurring during tumor growth, which suggests that targeting metabolic commensalism may serve as a therapeutic strategy [7].

Cancer stem cells (CSCs) are a small subset of cancer cells that are linked to self-renewal and are thus capable of driving sustained tumor growth, which resemble parental tumors on a histological and genetic level [8]. Over the past decade, studies have reported that CSCs are closely correlated with tumor initiation, metastasis and recurrence [9]. In a number of tumors including CRC, CSCs have been shown to exhibit long-term self-renewal and metastasis-initiating abilities [10, 11]. However, the mechanisms underlying the regulation of CSC metastasis remains poorly understood. The results of a previous study demonstrated that CSCs prefer to undergo OXPHOS rather than glycolysis [12]. Of note, OXPHOS is closely associated with acute myeloid leukemia stem cell self-renewal and survival [13]. Our previous study clearly showed that, in patient-derived organoids, the transfer of non-CSC-derived lactate to CSCs enhances OXPHOS activity, thus promoting CSC self-renewal in CRC [14]. In addition, it has been reported that circulating cancer cells exhibit enhanced OXPHOS activity compared with primary tumor cells [15], suggesting that alternative metabolic pathways in CSCs are closely associated with tumor metastasis.

Metastatic dissemination is a complex multistep process, and cancer cell intravasation through the blood vessel endothelium is considered to be the first step in the metastatic cascade [16]. Despite the reports of a previous study that an acidic TME is strongly associated with tumor metastasis [5], normoxic rather than hypoxic CSCs may get access to the circulatory system to cause distant metastasis due to being in well-vascularized areas [6]. Therefore, we hypothesized that TME-derived lactate may promote the metastatic potential of normoxic CSCs.

In the present study, the abundance of hypoxic and normoxic CSCs was analyzed using primary CRC tumor tissues and patient-derived xenograft tumors. Furthermore, using two human CRC cell lines (LoVo and SW480) and a patient-derived xenograft (XhCRC), the metabolic identities of normoxic CSCs at the cellular and molecular levels were investigated. Of note, the mechanisms through which TME-derived lactate promoted the metastatic potential of normoxic CSCs were explored. The present findings may provide a potential treatment strategy for tumor metastasis in patients with cancer, particularly CRC.

## Results

### The abundance of normoxic CSCs is positively associated with CRC tumor stage and metastasis

**Fig. 1:**
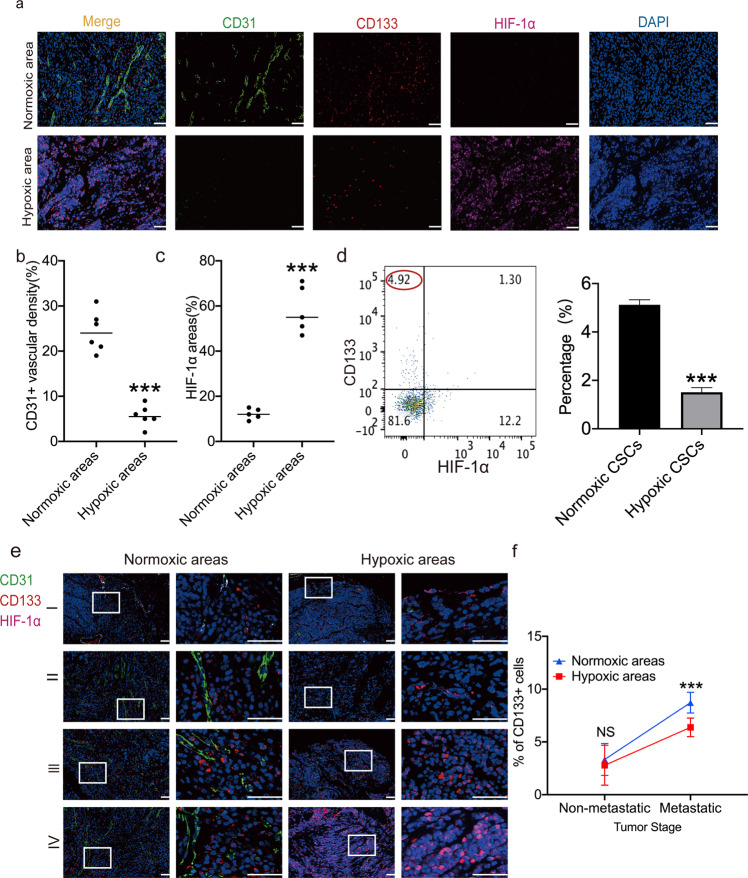
Abundance of normoxic CSCs is positively related to tumor stage of patients with CRC. **a** Representative immunofluorescence staining images of normoxic and hypoxic areas in primary CRC tumors. Scale bar: 50μm. **b** Quantification of vessel density in normoxic and hypoxic areas in primary CRC tumors (*n* = 6). **c** Quantification of ratios of HIF-1α^+^ tumor cells in normoxic and hypoxic areas in primary CRC tumors (*n* = 5). **d** Abundance of normoxic (i.e.CD133^+^HIF-1α^−^) and hypoxic CSCs (i.e. CD133^+^HIF-1α^+^) in primary CRC tumors by FACS analysis (*n* = 5). Experiments were repeated three times independently. **e** Representative immunofluorescence staining images in primary CRC tumors at different stages (i.e. TNM stage I (*n* = 9), II (*n* = 12), III (*n* = 10), IV (*n* = 21)). Scale bar: 50 μm. **f** Quantification analysis of abundance of CD133+ cells in normoxic and hypoxic areas in primary CRC tumors at non-metastatic (Stages I, II, III (*n* = 31)) versus metastatic (Stage IV (*n* = 21)) stages. Data are expressed as mean ± SD. *P < 0.05, **P < 0.01, ***P < 0.001.

To investigate CSC abundance in normoxic compartments of the TME (i.e. normoxic CSCs), immunofluorescence was performed to assess the expression of CD133, a widely used CSC marker [17], in a variety of tumor specimens from patients with CRC co-stained with HIF-1α to denote hypoxic regions [18] and CD31 to denote blood vessels [19]. The results revealed that the frequency of positive staining for HIF-1α (i.e. HIF-1α^+^ cells) was negatively correlated with that of positive staining for CD31 (i.e. CD31^+^ cells) in tumor stroma cells (Fig. 1a-c), indicating that TME blood vessels provide oxygen to tumor cells nearby. Further co-staining for CD133 and epithelial cellular adhesion molecule (an epithelial marker [20]), confirmed that all CD133^+^ cells were epithelial cells (Fig. S1a). To clarify the subpopulation of CSCs in a TME consisting of normoxic and hypoxic regions, primary CRC specimens were processed into single cells, according to our previous protocol [21, 22], in which the expression of CD133 and HIF-1α was then evaluated using flow cytometry. The results showed that, with regards to CD133 and HIF-1α expression, primary CRC cells were divided into 4 categories (Fig. 1d). Of note, the abundance of CD133^+^HIF-1α^−^ cells (i.e. normoxic CSCs) was higher than that of CD133^+^HIF-1α^+^ cells (hypoxic CSCs; Fig. 1d).

To confirm the role of normoxic CSCs in tumor progression, the abundance of CD133^+^HIF-1α^−^ cells in tumor specimens from patients with different stages of CRC was analyzed. Quantification analysis revealed that the abundance of CD133^+^HIF-1α^−^ was positively correlated with TNM stage (Fig. S1b and Fig. 1e), indicating that normoxic CSCs may contribute to CRC development. On the other hand, although the frequency of CD133^+^HIF-1α^+^ (i.e. hypoxic CSCs) was also elevated in CRC specimens with advanced stage CRC (Fig. S1c, and Fig. 1e), the CD133^+^HIF-1α^+^ cells were generally located far away from the CD31^+^ cells, suggesting that hypoxic CSCs have limited access to circulation and cannot easily form distant metastatic lesions (Fig. S1b and Fig. 1e). More importantly, in specimens from patients with stage IV CRC, the abundance of CD133^+^HIF-1α^−^ cells was higher than that of CD133^+^HIF-1α^+^ cells, indicating that normoxic CSCs may play a more important role in metastasis than hypoxic CSCs (Fig. 1e, f). In combination, these results suggested that normoxic CSCs are positively correlated with CRC development and metastasis.

### Lactate promotes the metastatic potential of normoxic CSCs

**Fig. 2:**
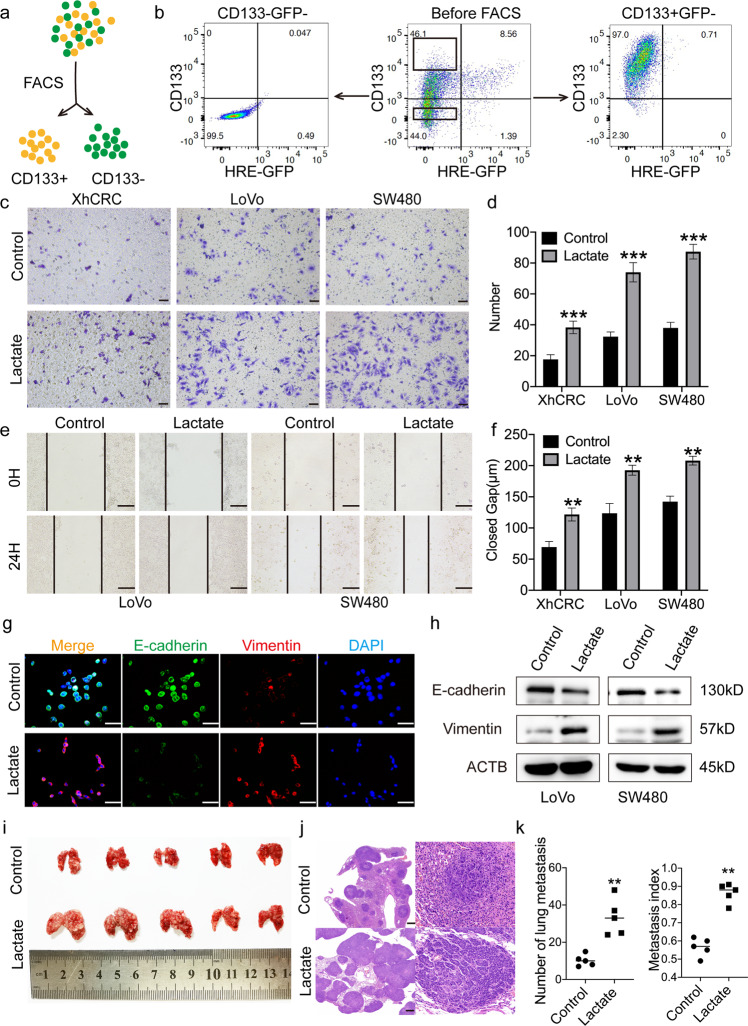
Lactate enhances metastatic potential of normoxic CSCs. **a** Schematic of CD133^+^GFP^−^ and CD133^−^GFP^−^ cell sorting. **b** An example of post-sorting analysis of purified CD133^+^GFP^−^ and CD133^−^GFP^−^ LoVo cells. Transwell invasion assays. XhCRC, LoVo and SW480 normoxic sphere-forming cells were proceeded into single cells and incubated with DMEM containing 5 mM lactate or control medium in 37 °C, 24 h later, invaded cells were photographed (**c**) and quantified (**d**). Scale bar: 200 μm. **e**, **f** Wound healing assays. XhCRC, LoVo and SW480 normoxic sphere-forming cells were cultured in the presence of 5 mM lactate for 24 h, DMEM/F12 as control. Representative images were shown in **e**. Quantified analysis was shown in **f**. Scale bar: 50 μm. **g** Representative immunofluorescence staining images of E-cadherin (green) and Vimentin (red) in LoVo normoxic sphere-forming cells treated with DMEM/F12 medium containing 5 mM lactate, DMEM/F12 medium as control. Nuclei were stained in blue. Scale bar: 50 μm. **h** Immunoblot analysis of E-cadherin and Vimentin in LoVo and SW480 normoxic sphere-forming cells treated with DMEM/F12 medium containing 5 mM lactate, DMEM/F12 medium as control. Loading control was assessed by ACTB (i.e. β-actin). **i**–**k** 1 × 10^6^ CD133^+^GFP^−^ SW480 cells treated with DMEM/F12 medium containing 5 mM lactate or DMEM/F12 medium as control were injected into tail vein of NOD/SCID mice (*n* = 5). Lactate treatment started 72 h pre injection. After 8 weeks, lung metastasis was determined. Representative images of lung metastasis were shown in i. H&E-stained lung sections were shown in **j**. Quantified analysis of metastatic lung nodules was shown in **k**. Scale bar: 500 μm. Data are expressed as mean ± SD. All in vitro experiments were repeated three times independently. *P < 0.05, **P < 0.01, ***P < 0.001.

Lactate is a TME-derived metabolic substrate for OXPHOS activity in tumor cells subject to normoxic conditions [6]. Furthermore, studies have reported that the accumulation of lactate in the TME may contribute to metastasis [4, 5]. In the present study, several experiments were conducted to investigate whether lactate promotes the metastatic potential of normoxic CSCs in the TME. First, a hypoxia-responsive element (HRE) driving the expression of green fluorescent protein (GFP) plasmid (p-HRE-GFP), which can faithfully record the hypoxic conditions of living cells [18], was transfected into various CRC cell lines, such as LoVo and SW480, and a patient-derived xenograft (XhCRC). The cells were further incubated with an anti-CD133 antibody in order to sort out the CD133^+^GFP^−^ cell population using flow cytometry (i.e. normoxic CSCs; Fig. 2a, b). The increased expression of several CSCs markers, such as CD133, Nanog and Sox2 [17], and low expression of HIF-1α in CD133^+^GFP^−^ cells were confirmed using immunoblotting assays (Fig. S2a). In addition, sphere-forming assays were performed for CRC cells under normoxic conditions (20% oxygen) to enrich normoxic CSCs [23, 24], and further confirmed by immunoblotting assays (Fig. S2a). Furthermore, we separated CD133^+^ and CD133^−^ CRC cells using flow cytometry and then co-stained with CD44 or CD26 [17]. The results showed that majority of CD133^+^ cells were double-positive cells (Fig. S2b). These results confirmed that purified CD133^+^cells did highly enrich for CSCs.

Next, in order to determine whether lactate promotes the metastatic potential of normoxic CSCs, purified CD133^+^GFP^−^ CRC cells were used to perform in vitro experiments, such as invasion and wound healing assays. The results demonstrated that, upon treatment with lactate, the invasive and migratory capacity of CD133^+^GFP^−^ cells was enhanced (Fig. 2c–f). To exclude the impact of lactate on tumor cell survival, cells were treated with lactate for 24 h, and cell apoptosis and proliferation were analyzed. Of note, treatment with lactate did not exert any significant effect on cell apoptosis or cell proliferation (Fig. S2c–e). Subsequently, in order to determine whether lactate promotes the migration and invasion of normoxic CSCs through epithelial-mesenchymal transition, immunofluorescence and immunoblotting analysis were performed for E-cadherin, a commonly used epithelial marker, and vimentin, a frequently used mesenchymal marker [25]. The results demonstrated that, upon treatment with lactate, the protein expression of E-cadherin was decreased and that of vimentin was increased in CD133^+^GFP^−^ cells (Fig. 2g, h), suggesting that lactate may promote the metastatic potential of normoxic CSCs. To validate the role of lactate in vivo, 1 × 10^6^ CD133^+^GFP^−^ SW480 cells pre-treated with lactate or control medium were injected into NOD/SCID mice though the tail vein. The results showed that the lactate-pre-treated cells formed more metastatic lesions when compared with control-pre-treated cells (Fig. 2i–k), suggesting that lactate promotes the metastatic potential of normoxic CSCs in vivo.

### TME-derived lactate enhances OXPHOS activity in normoxic CSCs

**Fig. 3:**
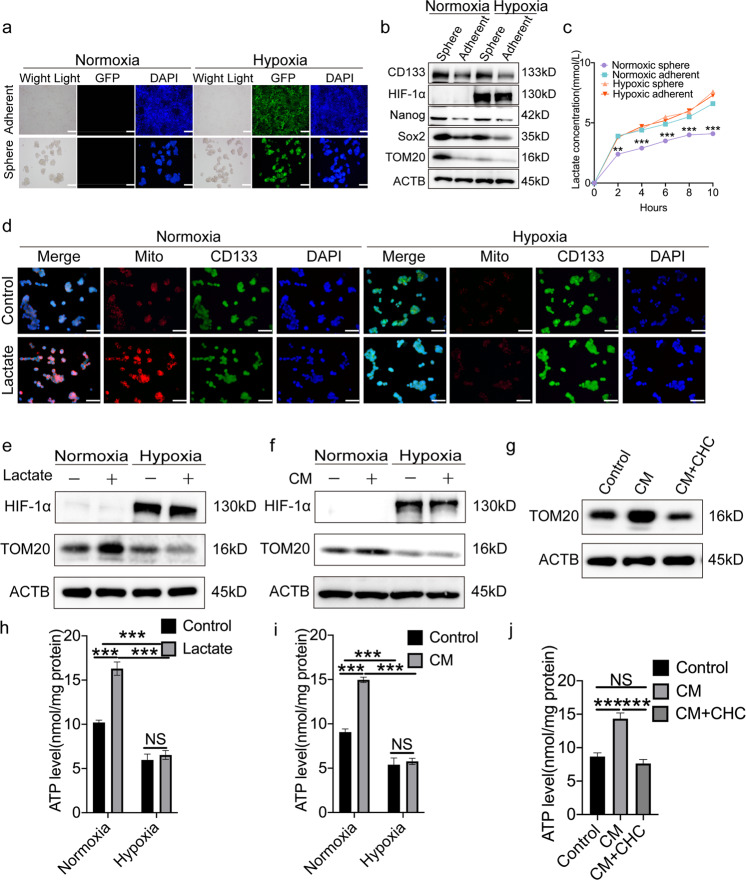
Lactate promotes OXPHOS of normoxic CSCs. **a** Representative immunofluorescence images of LoVo cells transfected with HRE reporter (p-HRE-GFP). Sphere and adherent-cultured LoVo cells were incubated in the medium under normoxic condition or in the medium containing CoCl_2_, respectively. Scale bar: 50 μm. **b** Immunoblot analysis of CD133, Nanog, Sox2, HIF-1α, and TOM20 in sphere and adherent-cultured SW480 cells incubated in the medium under normoxic condition or in the medium containing CoCl_2_. Loading control was assessed by ACTB (i.e. β-actin). **c** The abundance of lactate in the media of SW480 sphere-forming cells versus other tumor cell subsets were measured at the indicated time. **d** Representative immunofluorescence images of CD133 (green) and Mitotracker (red) staining in XhCRC sphere-forming cells treated with DMEM/F12 medium containing 5 mM lactate, DMEM/F12 medium as control. Nuclei were stained in blue. Cells were incubated under normoxic or hypoxic conditions, respectively. Scale bar: 50 μm. Immunoblot analysis of HIF-1α and TOM20 in SW480 normoxic sphere-forming cells treated with DMEM/F12 medium containing 5 mM lactate (**e**), conditioned medium (CM) (**f**), or CM plus 5 mM CHC (**g**), DMEM/F12 medium as control. Cells were incubated under normoxic or physical hypoxic conditions, respectively. Loading control was assessed by ACTB (i.e. β-actin). Intracellular ATP levels in SW480 normoxic sphere-forming cells treated with DMEM/F12 medium containing 5 mM lactate (**h**), conditioned medium (CM) (**i**), or CM plus 5 mM CHC (**j**), DMEM/F12 medium as control. Cells were incubated under normoxic or physical hypoxic conditions, respectively. Data are expressed as mean ± SD. All experiments were repeated three times independently. **P < 0.01, ***P < 0.001.

Tumor heterogeneity is an important feature of malignant tumors, and cancer cells with distinct metabolic phenotypes may positively interact to facilitate tumor progression [6]. To investigate OXPHOS activity among distinct subpopulations of tumor cells, normoxic and hypoxic tumor cells were established. First, CRC cell lines, including LoVo and SW480, and a patient-derived xenograft (XhCRC), were transfected with an HRE reporter (p-HRE-GFP). The cells were then further cultured in an incubator containing a normal (20%) or low (1%) oxygen level for >10 passages, as described in our previous study [20]. Fluorescence and immunoblotting confirmed that, under hypoxic conditions, the intensity of GFP (Fig. 3a) and the expression of HIF-1α (Fig. 3b) in both sphere-forming and adherent cells were elevated. In addition, immunoblotting demonstrated that the expression of CD133, Nanog and Sox2 was increased in sphere cells under either normoxic or hypoxic culture conditions, when compared with the same marker in adherent culture cells (Fig. 3b). Furthermore, as compared with hypoxic spheres or their corresponding adherent culture cells, the expression of translocase of outer membrane 20 (TOM20), a mitochondrial marker [14], was significantly increased in normoxic sphere-forming cells (Fig. 3b). At the same time, the lactate concentration in conditioned medium (CM) purified from normoxic sphere-forming cells was significantly lower than that in medium derived from other cell subsets (Fig. 3c). These results indicated that normoxic CSCs exhibit higher OXPHOS activity, while the corresponding hypoxic CSCs and differentiated cancer cells tend to undergo glycolysis.

Next, to clarify whether lactate enhances OXPHOS activity in normoxic CSCs, normoxic spheres were incubated with 5 mM lactate and underwent several experiments, including mitotracker staining, TOM20 immunoblotting and adenosine triphosphate (ATP) generation measurement. The results showed that incubation with lactate increased mitotracker staining intensity, TOM20 expression and ATP generation in normoxic sphere cells (Fig. 3d, e, h) when compared with hypoxic sphere cells, implying that lactate promotes OXPHOS activity in normoxic CSCs compared with hypoxic CSCs. Next, to determine whether glycolytic cell-derived secretion, such as those from differentiated cancer cells or hypoxic CSCs, could also enhance OXPHOS activity in normoxic CSCs, normoxic and hypoxic spheres were cultured in CM-derived cells undergoing glycolysis (Fig. 3f, i). The increased TOM20 expression and ATP generation in normoxic spheres suggested that glycolytic cell-derived CM could promote OXPHOS activity in normoxic CSCs (Fig. 3f, i). Of note, following treatment with α-cyano-4-hydroxycinnamate, a commonly used inhibitor of monocarboxylate transporter 1 (MCT1) that inhibits lactate uptake in cells [26], the promoting effect of CM was attenuated (Fig. 3g, j), suggesting that the lactate of glycolytic cell-derived CM may enhance OXPHOS activity in normoxic CSCs.

### Lactate oxidation promotes the migration and invasion of normoxic CSCs by maintaining OXPHOS activity

**Fig. 4:**
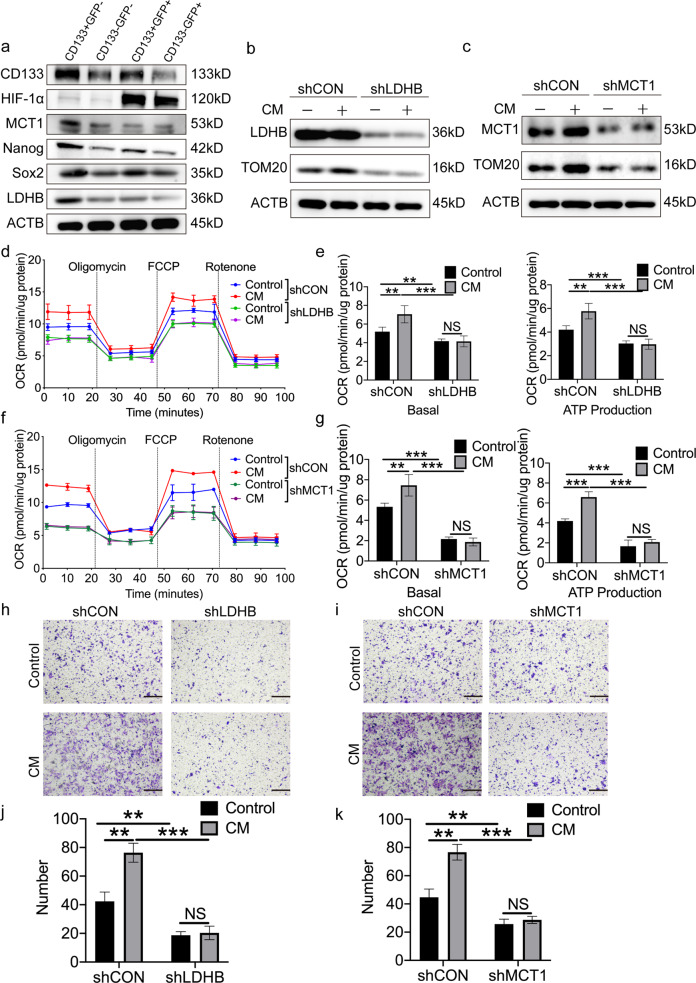
TME-derived lactate enhances migrative and invasive capacity of normoxic CSCs dependent on OXPHOs pathway. **a** Immunoblot analysis of CD133, Nanog, Sox2, HIF-1α, LDHB, and MCT1 in post-sorted SW480 cells. Cell hypoxia was induced under physical hypoxic conditions. Loading control was assessed by ACTB (i.e. β-actin). Immunoblot analysis of TOM20 in SW480 normoxic sphere-forming cells infected by LDHB (**b**) or MCT1 (**c**) shRNA lentivirus or vector. Cells were treated with CM for 24 h, DMEM/F12 medium as control. Loading control was assessed by ACTB (i.e. β-actin). **d**, **e** Seahorse analysis for oxygen consumption rate (OCR) of SW480 normoxic sphere-forming cells infected by LDHB shRNA lentivirus or vector. Cells were treated with CM for 24 h, DMEM/F12 medium as control. Quantification of the Seahorse data was shown in **e**. **f**, **g** Seahorse analysis for oxygen consumption rate (OCR) of SW480 sphere-forming cells infected by MCT1 shRNA lentivirus or vector. Cells were treated with CM for 24 h, DMEM/F12 medium as control. Quantification of the Seahorse data was shown in **g**. **h**–**k** Transwell invasion assays. SW480 normoxic sphere-forming cells were infected by MCT1 shRNA or LDHB shRNA lentivirus or vector. Cells were cultured in CM for 24 h, DMEM/F12 medium as control. Representative images were shown in **h** and **i**. Quantified analysis was shown in **j** and **k**. Scale bar: 200 μm. Data are expressed as mean ± SD. All experiments were repeated three times independently. *P < 0.05, **P < 0.01, ***P < 0.001.

The recycling and oxidation of TME-derived lactate is facilitated by MCT1, a transporter for exogenous lactate uptake [26], and lactate dehydrogenase B (LDHB), an enzyme that converts lactate into pyruvate for OXPHOS [27]. To investigate the molecular features of CSCs characterized by normoxic phenotypes, CRC cell lines, including LoVo and SW480, and a patient-derived xenograft (XhCRC) were first transfected with an HRE reporter, and further incubated with an anti-CD133 antibody to sort the CD133^+^GFP^−^ from other subpopulations of tumor cells using flow cytometry. An immunoblotting assay was then performed to evaluate the expression levels of MCT1 and LDHB in these cells. The results revealed that the protein expression of MCT1 and LDHB was increased in CD133^+^GFP^−^ cells, when compared with that in CD133^−^GFP^−^ or GFP^+^ cells (Fig. 4a). In addition, to further explore whether normoxic CSCs highly express MCT1 and/or LDHB, immunoblotting assays were performed for normoxic and hypoxic CRC sphere-forming cells, and adherent culture cells. The results showed that the expression of MCT1 and LDHB was elevated in normoxic sphere-forming cells, when compared with hypoxic sphere-forming cells or their corresponding adherent culture cells (Fig. S3a). These results clearly indicated that normoxic CSCs may be more efficient in consuming TME-derived lactate, when compared with either hypoxic CSCs or the corresponding non-CSCs. To clarify whether lactate oxidation affects the metabolic properties of normoxic CSCs, CRC cells were transfected with short hairpin (sh)RNA targeting the expression of MCT1 or LDHB. The reduced expression of MCT1 and LDHB was confirmed using immunoblotting analysis (Fig. 4b, c). The results showed that MCT1 and/or LDHB knockdown in normoxic sphere cells incubated with either CM or control medium resulted in decreased TOM20 expression and impaired oxygen consumption rates (OCR; Fig. 4b-g), suggesting that lactate oxidation contributes to the maintenance of OXPHOS activity in normoxic CSCs.

To clarify the role of lactate oxidation in the migration and invasion of normoxic CSCs, migration and invasion assays were performed using normoxic sphere-forming cells following MCT1 or LDHB knockdown. The results indicated that the invasion (Fig. 4h–k) and migration (Fig. S3b, c) of normoxic sphere-forming cells incubated with either CM or control medium were clearly attenuated following MCT1 or LDHB knockdown. Furthermore, LDHB or MCT1 knockdown had no effect on the apoptotic rate and proliferation of cells (Fig. S3d–i). To further elucidate whether lactate oxidation contributes to the migration of normoxic CSCs, normoxic sphere-forming cells were treated with rotenone, an aerobic respiratory inhibitor. These findings exhibited that the expression level of TOM20 was significantly decreased (Fig. S3j) in normoxic sphere-forming cells and that their migratory capacity was markedly reduced compared with that of the control (Fig. S3k, l). In combination, these data indicated that lactate oxidation promotes the migration and invasion of normoxic CSCs through the oxidation metabolic pathways.

### Peroxisome proliferator-activated receptor-γ coactivator-1α (PGC-1α) orchestrates lactate oxidation, thus promoting normoxic CSC metastasis

**Fig. 5:**
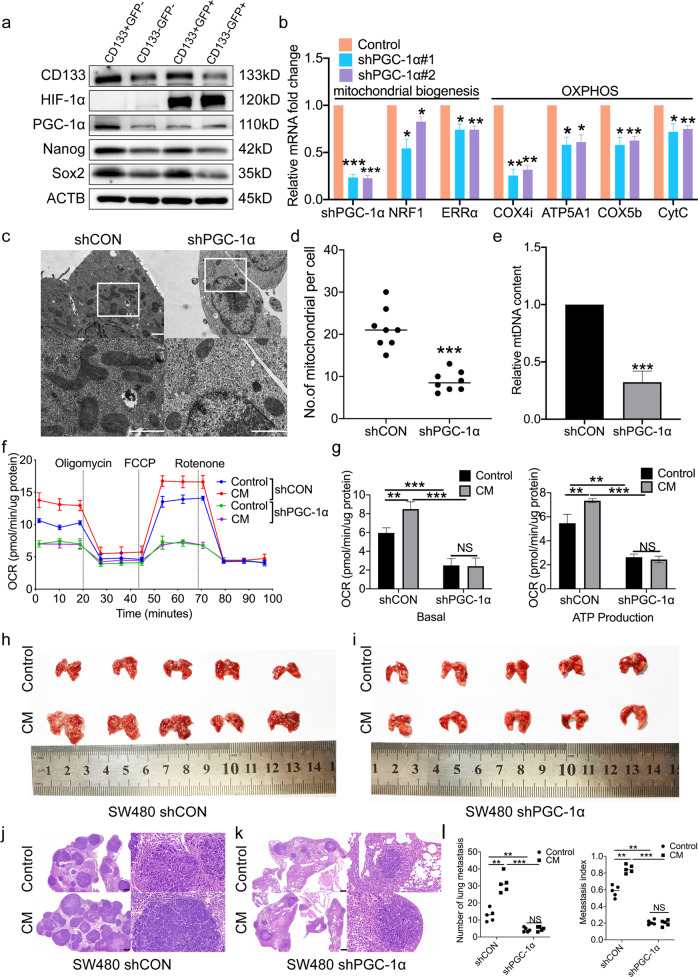
PGC-1α regulates mitochondrial biosynthesis and OXPHOS of normoxic CSCs. **a** Immunoblot analysis of CD133, Nanog, Sox2, HIF-1α, and PGC-1α in post-sorted SW480 cells. Cell hypoxia was induced under physical hypoxic conditions. Loading control was assessed by ACTB (i.e. β-actin). **b** RT-PCR analysis of mitochondrial biogenesis and oxidative phosphorylation genes expression in XhCRC normoxic sphere-forming cells infected by PGC-1α shRNA lentivirus or vector. **c**, **d** Electron microscopy of mitochondria number in SW480 sphere-forming cells infected by PGC-1α shRNA lentivirus or vector. Quantification of the number of mitochondria per cell in the imaged section (*n* = 8 cells) was shown in **d**. Scale bar: 1μm. **e** RT-PCR analysis of mitochondrial DNA content in SW480 sphere-forming cells infected by PGC-1α shRNA lentivirus or vector. **f**, **g** Seahorse analysis for oxygen consumption rate (OCR) of SW480 normoxic sphere-forming cells infected by. PGC-1α shRNA lentivirus or vector. Cells were treated with CM for 24 h, DMEM/F12 medium as control. Quantification of the Seahorse data was shown in **g**. **h**–**l** 1 × 10^6^ CD133^+^GFP^−^ SW480 cells infected by PGC-1α shRNA lentivirus or vector were injected into tail vein of NOD/SCID mice (*n* = 5). CM treatment started 72 h pre-injection, DMEM/F12 medium as control. After 8 weeks, lung metastasis was determined. Representative images of lung metastasis were shown in h and i. H&E-stained lung sections were shown in **j**, **k**. Quantified analysis of metastatic lung nodules was shown in **l**. Scale bar: 500 μm. Data are expressed as mean ± SD. All in vitro experiments were repeated three times independently. *P < 0.05, **P < 0.01, ***P < 0.001.

PGC-1α is a master regulator of mitochondrial biogenesis and activity [28, 29]. Our previous study demonstrated that CSCs are subject to confer mitochondrial OXPHOS activity [14]. Therefore, whether PGC-1α mediates lactate oxidation in normoxic CSCs was explored. The results showed that the expression of PGC-1α was significantly higher in normoxic sphere-forming cells than in the other tumor cell subsets (Fig. 5a). To clarify the role of PGC-1α in mediating cellular lactate oxidation, tumor cells were transfected with shRNA targeting the expression of PGC-1α (shPGC-1α), and a reduced PGC-1α expression was confirmed by RT-qPCR (Fig. 5b). It was found that, following PGC-1α knockdown, the mRNA expression of several genes associated with mitochondrial biogenesis (nuclear respiratory factor 1 and estrogen-related receptor alpha) and oxidative phosphorylation (cytochrome c oxidase subunit 4 isoform 1, mitochondrial, ATP synthase F1 subunit α, cytochrome C oxidase Subunit 5B and cytochrome c) was decreased in the cells (Fig. 5b). Furthermore, scanning electron microscopy revealed that, following PGC-1α knockdown, the number of mitochondria and mitochondrial DNA content in normoxic sphere-forming cells were decreased (Fig. 5c–e). Of note, PGC-1α knockdown resulted in a decreased cellular OCR levels in normoxic sphere-forming cells incubated with either CM or control medium (Fig. 5f, g). In combination, these results suggested that PGC-1α contributes to lactate oxidation in normoxic CSCs. To determine whether PGC-1α affects the migration and invasion of normoxic CSCs, several in vitro experiments were carried out. The results showed that the invasive (Fig. S4a, b) and migratory (Fig. S4c, d) abilities of normoxic sphere-forming cells incubated with either CM or control medium was significantly decreased following PGC-1α knockdown. Furthermore, PGC-1α knockdown had no effect on cell apoptosis and proliferation (Fig. S4e–g). To further explore whether lactate promotes the metastatic potential of normoxic CSCs through PGC-1α, normoxic shPGC-1α or shCON SW480 sphere-forming cells pre-treated with CM or control medium were injected into the tail vein of NOD/SCID mice. A total of 8 weeks later, normoxic shPGC-1α sphere cells had formed fewer metastatic lesions than shCON sphere cells in the lungs of the mice (Fig. 5h–l). Overall, these data clearly suggested that lactate promotes the metastatic potential of normoxic CSCs through PGC-1α.

### HIF-1α signaling is a negative regulator of PGC-1α-mediated lactate oxidation in normoxic CSCs

**Fig. 6:**
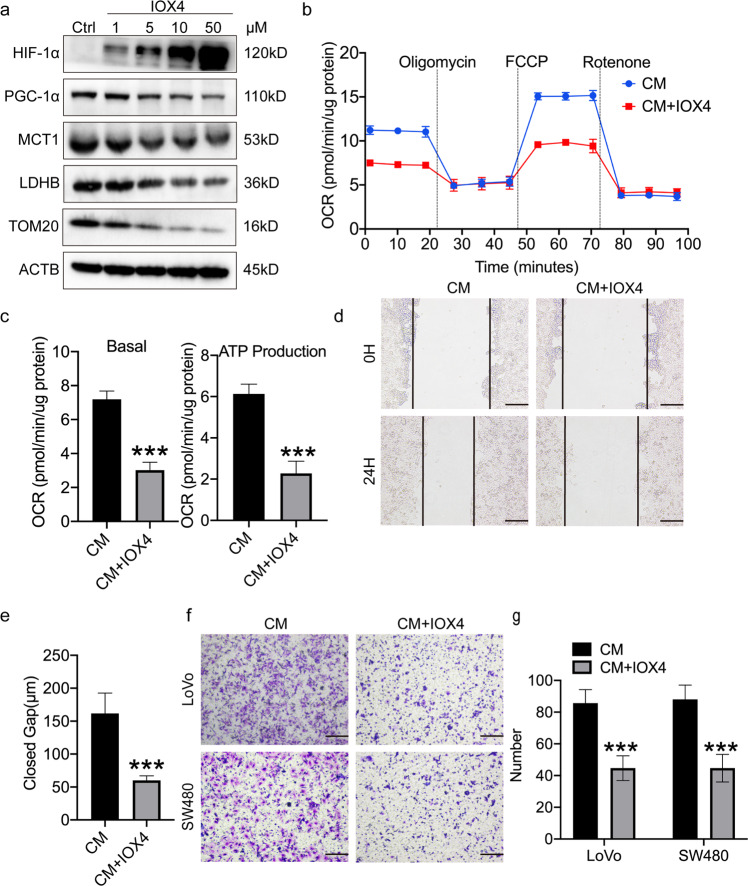
HIF-1α inhibits the expression of PGC-1α in hypoxic CSCs. **a** Immunoblot analysis of HIF-1α, PGC-1α, LDHB, MCT1, LDHB, and TOM20 SW480 normoxic sphere-forming cells treated by IOX4 or DMSO for 6 h in CM. Loading control was assessed by ACTB (i.e. β-actin). **b**, **c** Seahorse analysis for oxygen consumption rate (OCR) of SW480 normoxic sphere-forming cells treated by 50 μM IOX4 or DMSO for 6 h in CM. Quantification of the Seahorse data was shown in **c**. **d**, **e** Wound healing assays. SW480 normoxic sphere-forming cells were incubated with DMSO containing 50 μm IOX4 or DMSO for 6 h in CM. 24 h later, representative images of wound healing assay were photographed (**d**) and quantified (**e**). Scale bar: 200 μm. **f**, **g** Transwell invasion assays. LoVo and SW480 normoxic sphere-forming cells were proceeded into single cells and incubated with 50 μM IOX4 or DMSO for 6 h in CM in 37 °C, 24 h later, invaded cells were photographed (**f**) and quantified (**g**). Data are expressed as mean ± SD. All experiments were repeated three times independently. *P < 0.05, **P < 0.01, ***P < 0.001.

**Fig. 7:**
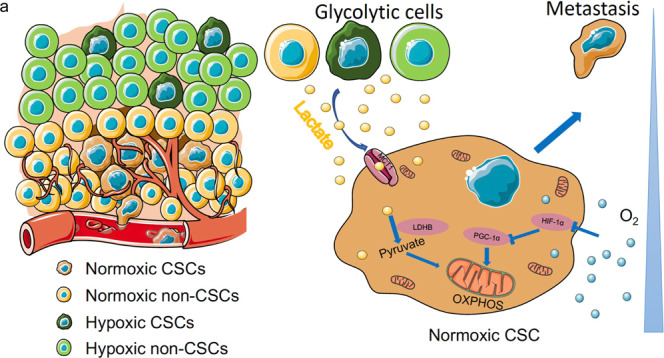
TME-derived lactate promotes metastasis of normoxic CSCs depending on oxidative metabolism pathway. **a** Schematic illustration of the proposed model. Normoxic CSCs get more access to the blood vessels than hypoxic CSCs. Hypoxic cell-secreted lactate in the TME could be ingested by normoxic CSCs to promote their OXPHOS and thus enhance their metastatic capacity.

A previous study has reported that the expression of PGC-1α is suppressed in clear cell renal cell carcinoma in an HIF-1α-dependent manner [30]. Consistent with this finding, the expression of HIF-1α was markedly reduced in normoxic sphere-forming cells (i.e. normoxic CSCs), when compared with hypoxic sphere-forming cells (Figs. 3b, 4a, and 5a). To explore how HIF-1α regulates PGC-1α in normoxic CSCs, a HIF-1α-inhibition model was first established, in which normoxic sphere-forming cells were treated a small-molecule inhibitor of prolyl hydroxylase domain 2, an enzyme that catalyzes the degradation of cellular HIF-1α [31]. As expected, HIF-1α expression was increased in normoxic sphere-forming cells following treatment with IOX4 for 6 h (Fig. 6a). Of note, IOX4-treated normoxic sphere-forming cells downregulated the expression of several lactate oxidation complex-related genes, including PGC-1α, MCT1, LDHB and TOM20. In addition, the effects of IOX4 on normoxic sphere-forming cells were dose-dependent (Fig. 6a). A decrease in OCR levels and ATP production was observed in IOX4-treated normoxic sphere-forming cells incubated with CM (Fig. 6b, c). Collectively, these data suggested that HIF-1α signaling negatively regulates PGC-1α-mediated lactate oxidation in normoxic CSCs. To elucidate whether HIF-1α signaling mediates the migration and invasion of normoxic CSCs, migration and invasion assays were performed for IOX4-treated normoxic CRC sphere-forming cells incubated with CM. The results revealed that treatment with IOX4 attenuated the migration and invasion of normoxic CSCs incubated with CM (Fig. 6d–g). In combination, the present findings suggested that HIF-1α signaling is a negative regulator of PGC-1α-mediated lactate oxidation in normoxic CSCs, and may thus promote the metastasis of CSCs located in normoxic regions in CRC tumors (Fig. 7a).

## Discussion

TME, a dynamic system orchestrated by intercellular communications, has emerged as an important contributor to tumor progression and metastasis [32]. The TME contains various cells, such as CSCs, differentiated cancer and stromal cells, as well as metabolic byproducts such as lactate [33]. CSCs reside in the TME and have been reported to be responsible for tumor recurrence and metastasis [8]. Recent studies have shown that the components of the TME interact with CSCs, thus impacting the metastatic capacity of CSCs [34]. Hypoxia occurs frequently in human solid tumors, including CRC. Due to rapid growth, CRC tumor tissue suffers from hypoxia, as the tumor vasculature is unable to supply it with enough oxygen and nutrients [35]. Therefore, tumor cells become increasingly hypoxic as their distance from a functional blood supply increases. Despite previous studies suggesting that tumors growing in hypoxic environments are more prone to rapid growth and invasion, it is difficult for hypoxic tumor cells to enter the blood vessels and form distant metastatic lesions. As a result of hypoxia, tumor cells produce metabolic byproducts, such as lactate [36]. Recent studies have indicated that lactate is potentially an important molecule and energy source of TME acidification and leads to an increase in the motility of cancer cells [37, 38]. More importantly, lactate is positively associated with tumor metastasis and recurrence [39]. Consistent with the other studies, our previous study showed that differentiated cancer cell-derived lactate promotes the self-renewal of CSCs in CRC [14]. Of note, another previous study has demonstrated that hypoxic and normoxic cells exist in colorectal tumor tissues due to uneven blood supply, and that hypoxic CRC cells promote the metastasis of normoxic CRC cells [18]. In the present study, using primary tumors and cells derived from patients with CRC, the existence of normoxic CSCs was revealed, and the abundance of normoxic CSCs was found to be positively correlated with tumor stage. More interestingly, using CRC cell lines and patient-derived xenograft cells, TME-derived lactate was found to enhance OXPHOS activity, as well as the invasive, migratory and metastatic abilities of normoxic, but not hypoxic, CSCs.

Intratumor heterogeneity among cancer cells can arise through multiple means. One of the mechanisms through which tumorigenic CSCs differentiate into nontumorigenic cancer cells may lead to a hierarchical organization within tumors. In addition, microenvironmental cues also contribute to the heterogeneity of tumor cancer cells in different locations, which stems largely from vascular integrity and proximity to the vasculature that create oxygen and nutrient gradients. Consequently, tumor cells in different locations exhibit distinct metabolic profiles [3, 6]. We therefore speculated that there may exist metabolic differences between normoxic and hypoxic CSCs. Studies have shown that, in non-small cell lung cancer, tumor cells in less-perfused regions (i.e. hypoxic cells) undergo glycolysis, while well-vascularized tumor cells (i.e. normoxic cells) utilize multiple nutrients, including lactate for OXPHOS [38]. This emerging concept of the “reverse Warburg effect” has been observed in a variety of tumors and received considerable attention in recent years [40]. It has been shown that the enhanced oxidative metabolism of tumor cells is associated with chemoresistance and metastasis [6]. Of note, tumor cells with a distinct metabolic identity in different regions cooperate to form metabolic “symbiosis”. For example, hypoxic tumor cells undergo anaerobic glycolysis and generate lactate, which is then taken up and used as a fuel for OXPHOS by normoxic tumor cells in adjacent oxygenated regions [41]. This metabolic crosstalk between tumor cells occurs in various solid tumors and has been proven to be critical to tumor development [7]. Consistent with these studies, the present results found that most hypoxic CSCs existed in hypoperfused regions, and normoxic CSCs were mainly located in well-vascularized regions of primary CRC tumor tissue. In addition, the present findings clearly illustrated that normoxic CSCs have a higher mitochondrial activity than hypoxic CSCs and undergo OXPHOS. Of note, TME-derived lactate enhances OXPHOS activity in normoxic CSCs, which in turn promotes their metastatic capacity. On the contrary, hypoxic CSCs are highly dependent on glycolysis and do not exhibit a response to lactate treatment.

An abundance of lactate is a common feature of TME in various solid tumors, including CRC [3]. Lactate uptake is dependent on MCT1 [26], and LDHB is responsible for converting lactate into pyruvate for OXPHOS [27]. It has been reported that a high expression of MCT1 and LDHB is positively correlated with an enhanced metastatic capacity in several malignancies [42, 43]. Consistent with these findings, the present study found that, compared with hypoxic CSCs, normoxic CSCs exhibited a much higher MCT1 and LDHB expression. Furthermore, MCT1 and LDHB knockdown inhibited OXPHOS activity, thus decreasing the migratory and invasive capacity of normoxic CSCs in vitro.

PGC-1α is a master regulator of mitochondrial biogenesis and plays a central role in regulating energy homeostasis and metabolism [44]. Recent studies have shown that the expression level of PGC-1α varies considerably in different tumor cells. Those cells with a high expression of PGC-1α generally tend to undergo OXPHOS [45, 46]. In addition, PGC-1α plays a crucial role in promoting tumor metastasis [15]. Consistent with previous studies, the present results indicated that normoxic CSCs exhibited high levels of PGC-1α expression and increased mitochondrial activity in response to lactate treatment compared with hypoxic CSCs. Furthermore, decreased OXPHOS activity was observed in normoxic CSCs following PGC-1α silencing. Of note, the metastatic capacity of normoxic CSCs was significantly reduced in response to PGC-1α knockdown.

The reason why PGC-1α expression varies in normoxic and hypoxic CSCs remains unclear, but hypoxia may be a key factor. In response to hypoxia, tumor cells set off dynamic adaptive responses through the stabilization and activation of HIFs and HIF-1 signaling pathways [47]. HIF-1α regulates the biological behavior of tumors by mediating tumor metabolism by inducing glycolysis [48, 49]. Of note, studies have shown that basic-helix-loop-helix transcription factor deleted in esophageal cancer 1, a hypoxia-inducible gene that acts as a transcriptional repressor, acts downstream of HIF stabilization to functionally suppress PGC-1α in clear cell RCC [30]. Another study has revealed that neuronal PAS domain protein 2 promotes aerobic glycolysis of hepatocellular carcinoma cells through the transcriptional upregulation of HIF-1α, thus promoting PGC-1α downregulation [50]. In the present study, it was demonstrated that increased HIF-1α expression leads to a decrease in PGC-1α expression, thus reducing OXPHOS activity in normoxic CSCs. Therefore, these findings illustrated that HIF-1α plays a negative role in maintaining mitochondrial activity in normoxic CSCs.

In conclusion, the results of the present study demonstrated that normoxic CSCs, which are located closer to the tumor-related vessels than hypoxic CSCs, undergo OXPHOS by ingesting lactate produced in the TME, thus achieving enhanced metastatic capacity. The present data suggested that effective therapies targeting the oxidative metabolism of normoxic CSCs may be a potential therapeutic approach to reducing metastasis in patients with cancer, particularly CRC.

## Materials and methods

### Antibody and reagents

See the Supplementary Materials and methods for the details.

### Cell lines and cell culture

The LoVo and SW480 human colon cancer cell lines were purchased from cell bank of Chinese Academy of Sciences (Shanghai, China). XhCRC cells were obtained and isolated from human CRC xenograft tumors as previously described [21].

Sphere-forming assay and adherent culture were performed as previously described [23], see the Supplementary Materials and methods for more details.

### Collection of primary CRC tissue and single cell

Primary single cells were obtained as previously described [21, 22]. All human subjects studies were obtained with patients’ informed consent and performed under the guidelines and protocols approved by the ethical committe of Tongji Hospital, Tongji Medical College, HUST (IRB ID: 20141106). The clinic information is listed in Supplementary Table 1. See the Supplementary Materials and methods for more details.

### Hypoxia treatment

Two methods were performed to obtain hypoxic tumor cells as previously described [18]. See the Supplementary Materials and Methods for more details.

### Collection of normoxic and hypoxic cancer stem cells

Two methods were used to collect normoxic and hypoxic cancer stem cells. See the Supplementary Materials and methods for more details.

### Collection of conditioned medium

The conditioned medium was derived from hypoxic CSCs and differentiated cells. CM was collected when cell cultures reached 90% confluency as as previously described [18]. See the Supplementary Materials and methods for more details.

### Stable transfection of PGC-1a, LDHB, and MCT1 shRNA

See the Supplementary Materials and methods for more details.

### Flow cytometry

Flow cytometry data were acquired by using a FACS Aria II Cell Sorter (BD Biosciences, San Jose, CA, USA), followed by flow cytometric analysis using Diva software (BD Biosciences). See the Supplementary Materials and methods for more details.

### Invasion and wound healing assays

The migration and invasion capacity of tumor cells were determined by wound healing assay and transwell system using 24-well plates with 8-μm transwell inserts (Corning, Inc., Corning, NY, USA). See the Supplementary Materials and Methods for more details.

### Tissue immunofluorescence

Tissue immunofluorescence staining was performed in paraffin-embedded CRC specimens. See the Supplementary Materials and methods for more details.

### Cell immunofluorescence

See the Supplementary Materials and methods for the details.

### Real-time PCR

See the Supplementary Materials and methods for the details.

### Western blot analysis

See the Supplementary Materials and methods for the details.

### Measurement of oxygen consumption rate

The mitochondrial oxygen consumption rate (OCR) was determined by using the Seahorse XF Cell Mito Stress Test Kit (Agilent Technologies Inc, CA, USA, 103015-100) and Seahorse XF Cell Mito Stress Test Starter Pack (Agilent Technologies Inc, 102601-100) according to the manufacturer’s instructions. See the Supplementary Materials and methods for the details.

### ATP measurements

ATP measurements were obtained using the ATP Determination kit (Life Technologies) according to the manufacturer’s instructions. See the Supplementary Materials and methods for the details.

### Cell counting kit-8 (CCK8) assay

CCK8 (MedChemExpress) was used to assess cell proliferation. See the Supplementary Materials and methods for the details.

### In vivo mouse assays

All animal studies were performed under the guidelines and protocols approved by the Institutional Animal Care and Use Committee of Tongji Medical College, Huazhong University of Science and Technology (IACUC ID:2014S652). Details are described in the Supplementary Materials and methods.

### Statistical analyses

All data were normalized to control and presented as means ± SEM. Significance was determined by One-way ANOVA or unpaired two-tailed Student’s *t* test and *p* < 0.05 was considered statistically significant. Analyses were conducted using SPSS version 24.0 (IBM, Armonk, NY, USA).

## Supplementary information

Supplementary Figures

Supplementary Table 1

Supplementary Table 2

Supplementary Materials and Methods

Original western blots

aj-checklist

## Data Availability

The datasets used and analyzed during this study are available from the corresponding author on reasonable request.
