# Supplementary material for: Lactate promotes metastasis of normoxic colorectal cancer stem cells through PGC-1α-mediated oxidative phosphorylation
Source: Cell Death Dis. 2022 Jul 27;13(7):651. doi: 10.1038/s41419-022-05111-1 (PMC9329320; doi:10.1038/s41419-022-05111-1)
Supplement: Supplementary file 1 — Supplementary Figures [file 41419_2022_5111_MOESM1_ESM.pdf]

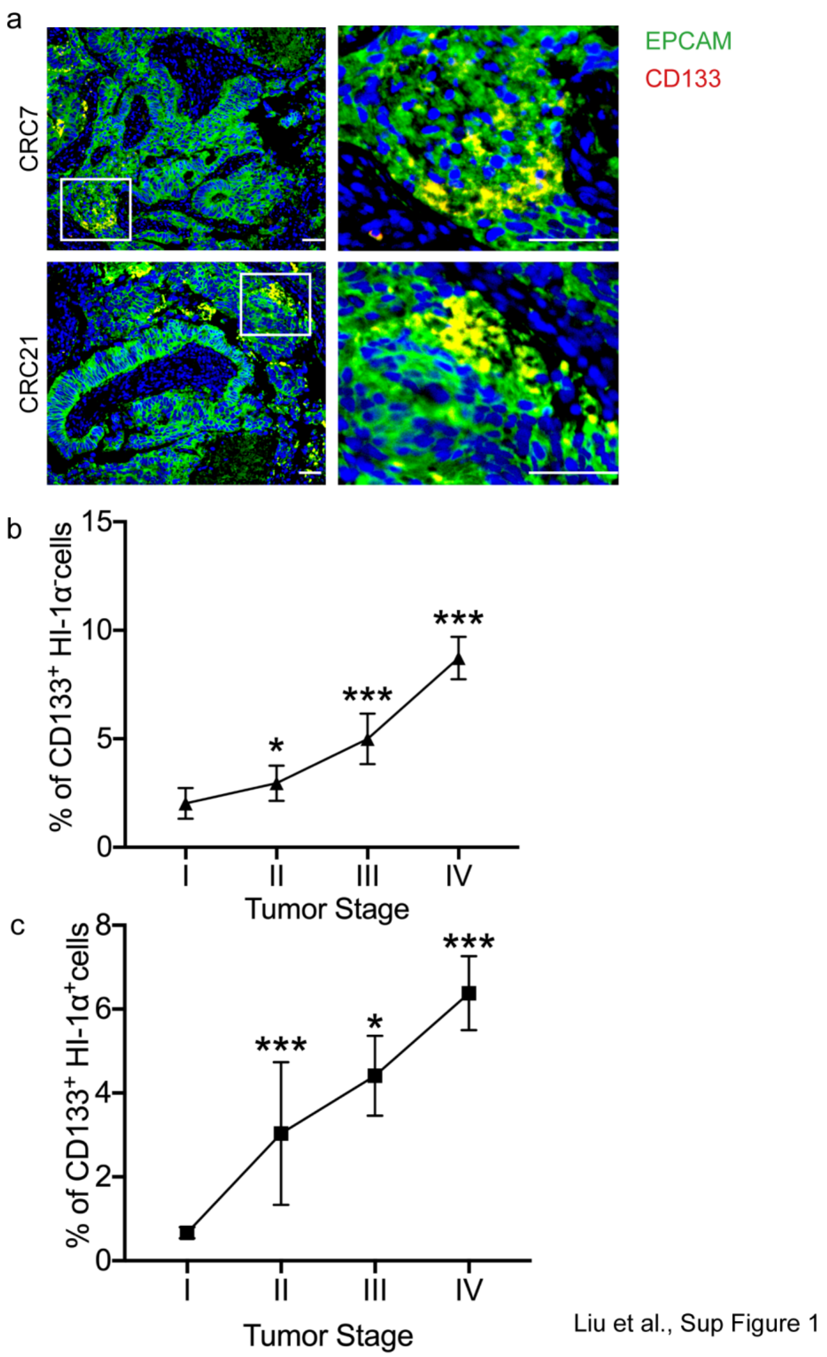

Supplementary Figure 1. Normoxic and Hypoxic CSCs in primary CRCs

a. Representative immunofluorescence staining images of EPCAM (green) and CD133(red) in primary CRC tumors. Scale bar: 50μm. B-c. Quantified analysis of the percentage of normoxic CSCs (i.e. $CD133^{+}HIF\text{-}1\alpha^{-}$) in primary CRC tumors at different tumor stages(i.e. TNM stage I(n=9), II(n=12), III(n=10), IV(n=21)). Scale bar: 50μm. c. Quantified analysis of the percentage of hypoxic CSCs (i.e. $CD133^{+}HIF\text{-}1\alpha^{+}$) in primary CRC tumors at different tumor stages(i.e. TNM stage I(n=9), II(n=12), III(n=10), IV(n=21)). Scale bar: 50μm. Data are expressed as mean ± SD. $*P < 0.05$, $**P < 0.01$, $***P < 0.001$.

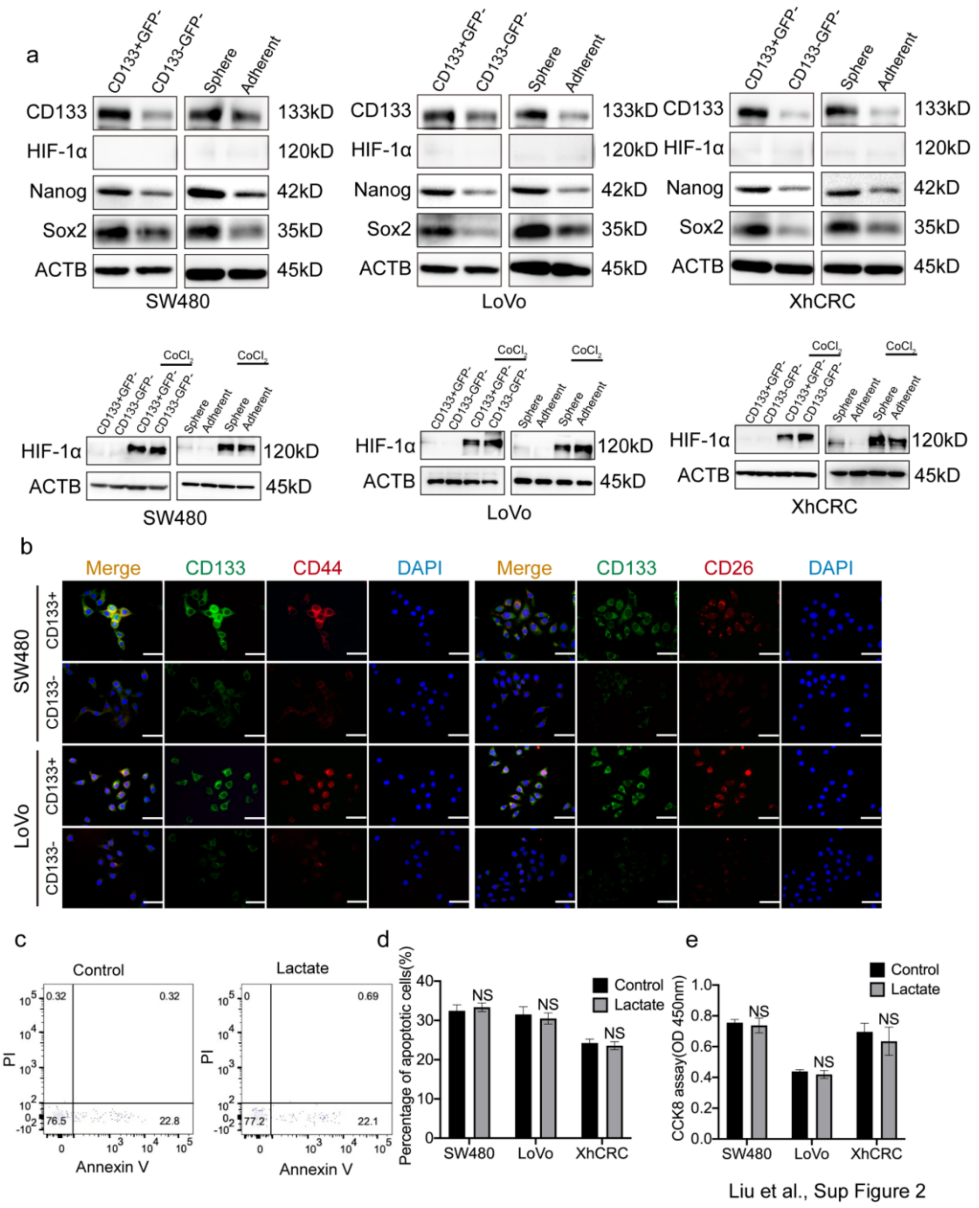

Supplementary Figure 2. Normoxic CSCs obtained ex vivo

a. Immunoblot analysis of CD133, Nanog, Sox2 and HIF-1α in post-sorted or sphere-forming SW480, LoVo and XhXRC cells. $CoCl_2$ treatment(300μM) was used as a positive control for HIF-1α expression. Loading control was assessed by ACTB (i.e. β-actin). b. Representative immunofluorescence staining images of CD133 (green) and CD44 (red) or CD26 (red) in LoVo and SW480 cells. Nuclei were stained in blue. Scale bar: 50μm. c. FACS analysis of apoptosis of XhCRC normoxic sphere-forming cells treated in DMEM/F12 medium containing 5mM lactate,DMEM/F12 medium as control. d. Statistical analysis of cell apoptosis via flow cytometry. e. Cell proliferation was detected by CCK8 analysis. Data are expressed as mean ± SD. All experiments were repeated three times independently. $*P < 0.05$, $**P < 0.01$, $***P < 0.001$.

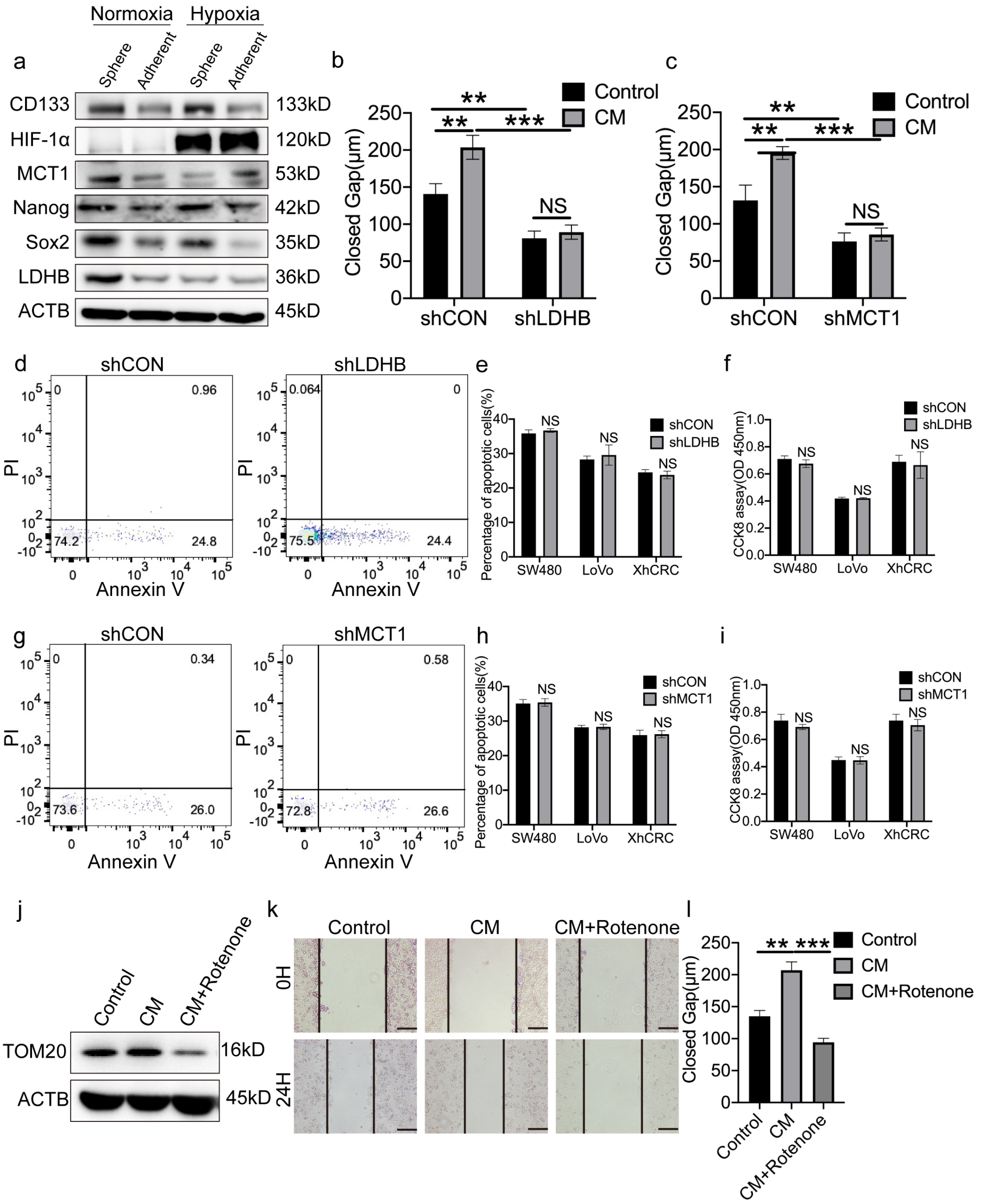

Liu et al., Sup Figure 3

Supplementary Figure 3. Inhibition of lactate uptake affects invasive and migratory capacity of normoxic CSCs dependent on OXPHOs pathway.

a. Immunoblot analysis of CD133, HIF-1α, Nanog, Sox2, LDHB and MCT1 in sphere and adherent-cultured SW480 cells incubated under normoxic or $CoCl_2$ induced hypoxic conditions. Loading control was assessed by ACTB (i.e. β-actin). b-c. Wound healing assays. SW480 normoxic sphere-forming cells were infected by LDHB shRNA or MCT1 shRNA lentivirus or vector. Cells were cultured with CM for 24 hours, DMEM/F12 medium as control. d. FACS analysis of apoptosis of

XhCRC normoxic sphere-forming cells infected by LDHB shRNA lentivirus or vector. e. Statistical analysis of cell apoptosis via flow cytometry. f. Cell proliferation was detected by CCK8 analysis. g. FACS analysis of apoptosis of XhCRC normoxic sphere-forming cells infected by MCT1 shRNA lentivirus or vector. h. Statistical analysis of cell apoptosis via flow cytometry. i. Cell proliferation was detected by CCK8 analysis. j. Immunoblot analysis of TOM20 in SW480 normoxic sphere-forming cells treated with 5μM rotenone, DMSO as control. Loading control was assessed by ACTB (i.e. β-actin). k-l. Wound healing assays. Cells were cultured in CM for 24 h, DMEM/F12 medium as control. SW480 normoxic sphere-forming cells were treated with DMSO containing 5μm rotenone or DMSO. Cells were cultured with CM for 24 hours, DMEM/F12 medium as control. 24h later, representative images were photographed (k) and quantified (l). Data are expressed as mean ± SD. All in vitro experiments were repeated three times independently. $*P < 0.05$, $**P < 0.01$, $***P < 0.001$.

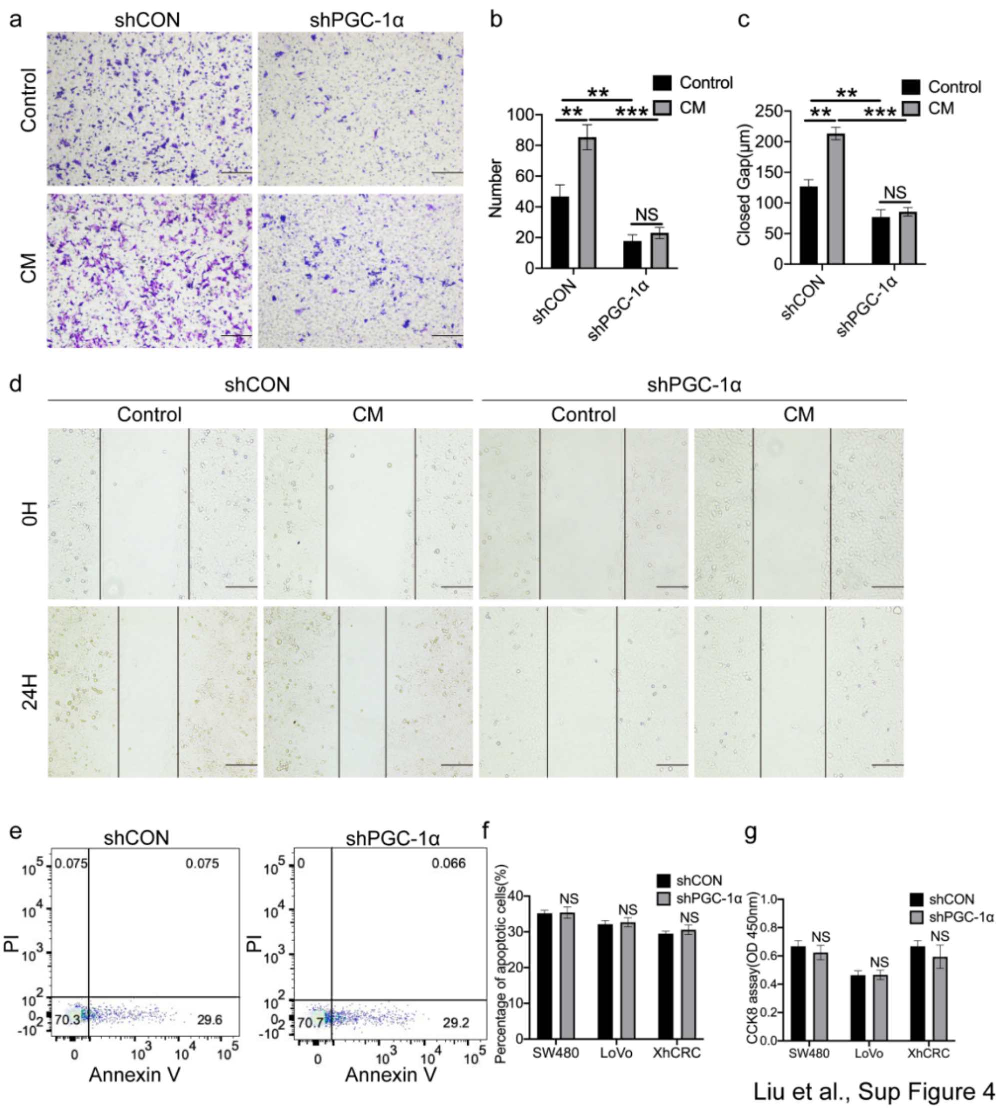

Supplementary Figure 4. PGC-1α knockdown impairs invasive and migratory capacity of normoxic CSCs.

a-b. Transwell invasion assays. SW480 normoxic sphere-forming cells were infected by PGC-1α shRNA lentivirus or vector. Cells were cultured in CM for 24 h, DMEM/F12 medium as control. Representative images were shown in a. Quantified analysis was shown in b. c-d. Wound healing assays. SW480 normoxic sphere-forming cells infected by PGC-1α shRNA lentivirus or vector were cultured with CM for 24 hours, DMEM/F12 medium as control. 24h later, representative images were photographed (c) and quantified (d). e. FACS analysis of apoptosis of XhCRC normoxic sphere-forming cells infected by PGC-1α shRNA lentivirus or vector. f. Statistical analysis of cell apoptosis via flow cytometry. g. Cell proliferation was detected by CCK8 analysis. Data are expressed as mean ± SD. All experiments were repeated three times independently. $^*P < 0.05$, $^{**}P < 0.01$, $^{***}P < 0.001$.
