# Supplementary material for: Lactate promotes metastasis of normoxic colorectal cancer stem cells through PGC-1α-mediated oxidative phosphorylation
Source: Cell Death Dis. 2022 Jul 27;13(7):651. doi: 10.1038/s41419-022-05111-1 (PMC9329320; doi:10.1038/s41419-022-05111-1)
Supplement: Supplementary file 2 — Supplementary Table 1 [file 41419_2022_5111_MOESM2_ESM.docx]

Supplementary Table 1. Clinical history of human subjects.

| Patient No. | Gender | Age | TNM | Tumor Stage |
| --- | --- | --- | --- | --- |
| CRC1 | M | 63 | pT2N0M0 | I |
| CRC2 | M | 48 | pT1N0M0 | I |
| CRC3 | M | 62 | pT3N0M0 | II |
| CRC4 | F | 57 | pT3N1aM0 | III |
| CRC5 | F | 65 | pT2N0M0 | I |
| CRC6 | M | 63 | pT4bN1M1 | IV |
| CRC7 | M | 47 | pT3N0M0 | II |
| CRC8 | M | 64 | pT3N0M0 | II |
| CRC9 | F | 67 | pT3N1M0 | III |
| CRC10 | F | 67 | pT3N1cM0 | III |
| CRC11 | M | 58 | pT3N1cM0 | III |
| CRC12 | M | 36 | pT3N0M0 | II |
| CRC13 | F | 58 | pT3N0M0 | II |
| CRC14 | M | 61 | pT4N0M1 | IV |
| CRC15 | F | 52 | pT2N0M0 | I |
| CRC16 | M | 57 | pT3N0M0 | II |
| CRC17 | M | 50 | pT3N1M0 | III |
| CRC18 | M | 67 | pT1N0M1 | IV |
| CRC19 | M | 71 | pT3N1bM0 | III |
| CRC20 | F | 69 | pT3N0M0 | II |
| CRC21 | M | 74 | pT1N0M0 | I |
| CRC22 | M | 48 | pT4N1M1 | IV |
| CRC23 | M | 65 | pT3N0M0 | II |
| CRC24 | M | 64 | pT3N1cM1 | IV |
| CRC25 | F | 58 | pT3N1cM0 | III |
| CRC26 | F | 64 | pT4N2M1 | IV |
| CRC27 | F | 50 | pT3N0M0 | II |
| CRC28 | M | 64 | pT3N0M1 | IV |
| CRC29 | F | 58 | pT3N2M1 | IV |
| CRC30 | M | 61 | pT3N0M0 | II |
| CRC31 | M | 70 | pT2N0M0 | I |
| CRC32 | F | 48 | pT3N2M0 | III |
| CRC33 | F | 51 | pT3N0M0 | II |
| CRC34 | M | 53 | pT3N0M0 | II |
| CRC35 | M | 70 | pT4N0M1 | IV |
| CRC36 | M | 44 | pT1N0M0 | I |
| CRC37 | F | 51 | pT2N0M0 | I |
| CRC38 | F | 52 | pT2N0M0 | I |
| CRC39 | F | 66 | pT4N2M1 | IV |
| CRC40 | M | 50 | pT4aN2bM0 | III |
| CRC41 | F | 64 | pT3N2bM0 | III |
| CRC42 | M | 67 | pT3N0M1 | IV |
| CRC43 | F | 62 | pT3N1M1 | IV |
| CRC44 | M | 50 | pT4N1cM1 | IV |
| CRC45 | F | 49 | pT4N0M1 | IV |
| CRC46 | M | 65 | pT3N1M1 | IV |
| CRC47 | M | 69 | pT4N1M1 | IV |
| CRC48 | F | 57 | pT3N1M1 | IV |
| CRC49 | M | 56 | pT3N0M1 | IV |
| CRC50 | M | 65 | pT3N2M1 | IV |
| CRC51 | F | 52 | PT4N1M1 | IV |
| CRC52 | M | 70 | PT3N2M1 | IV |
