# Supplementary material for: Lactate promotes metastasis of normoxic colorectal cancer stem cells through PGC-1α-mediated oxidative phosphorylation
Source: Cell Death Dis. 2022 Jul 27;13(7):651. doi: 10.1038/s41419-022-05111-1 (PMC9329320; doi:10.1038/s41419-022-05111-1)
Supplement: Supplementary file 3 — Supplementary Table 2 [file 41419_2022_5111_MOESM3_ESM.docx]

Supplementary Table 2. Sequence of the primers used for RT-qPCR.

| Genes | Sequence **(5’ to 3’)** | |
| --- | --- | --- |
| PGC1α | forward | TCTGAGTCTGTATGGAGTGACAT |
|  | reverse | CCAAGTCGTTCACATCTAGTTCA |
| ERRα | forward | GAGATCACCAAGCGGAGACG |
|  | reverse | ATGAGACACCAGTGCATTCAC |
| Cox5b | forward | ATGGCTTCAAGGTTACTTCGC |
|  | reverse | CCCTTTGGGGCCAGTACATT |
| Cytc | forward | CTTTGGGCGGAAGACAGGTC |
|  | reverse | TTATTGGCGGCTGTGTAAGAG |
| NRF1 | forward | AGGAACACGGAGTGACCCAA |
|  | reverse | TGCATGTGCTTCTATGGTAGC |
| ATP 5a1 | forward | TGCAAGGAACTTCCATGCCTC |
|  | reverse | CGCCCAGTTTCTTCAAGATCAA |
| ACTB | forward | AGGGAAATCGTGCGTGACAT |
|  | reverse | CGTTGCCAATAGTGATGACC |
| mtCO1 | forward | TGGAGCCTCCGTAGACCTAA |
|  | reverse | TGCGAAGCCTGGTAGGATAA |
| Ndufv1 | forward | CTCCCCAGCCGCCTCAAG |
|  | reverse | CCAACCCCCAGGATCAGCAGC |
