# Supplementary material for: Lactate promotes metastasis of normoxic colorectal cancer stem cells through PGC-1α-mediated oxidative phosphorylation
Source: Cell Death Dis. 2022 Jul 27;13(7):651. doi: 10.1038/s41419-022-05111-1 (PMC9329320; doi:10.1038/s41419-022-05111-1)
Supplement: Supplementary file 4 — Supplementary Materials and Methods [file 41419_2022_5111_MOESM4_ESM.docx]

**1. Supplementary Materials and Methods**

**1.1. Antibody and reagents**

The antibodies used in the present study included: Rabbit anti human CD133(Cell Signaling Technology, Danvers, MA, USA, 64326), rabbit anti human Sox2 (Cell Signaling Technology, 3579), rabbit anti human Nanog (Cell Signaling Technology, 4903), rabbit anti human HIF-1α (Cell Signaling Technology, 36169), rabbit anti human EPCAM (ABclonal, Wuhan, China, A19301), rabbit anti human CD31 (ABclonal, A19014), rabbit anti human PGC-1α (ABclonal, A10891), rabbit anti human LDHB (ABclonal, A7625), rabbit anti human MCT4 ( Proteintech, Wuhan, China, 22787-1-AP), rabbit anti human MCT1 (abcam, Cambridge, UK, ab90582), rabbit anti human ACTB (ABclonal, AC026), rabbit anti human TOM20 (Cell Signaling Technology, 42406); APC-anti‐human EPCAM(Biolegend, San Diego, CA, USA, 324208); PE‐anti‐human CD133(Biolegend, 372804); 488-conjugated-anti-rabbit IgG(Cell Signaling Technology, 12935).

**1.2. Cell lines and cell culture**

For sphere-forming assay, 1 × 10^5^ tumor cells were cultured on ultra-low attachment 6-well culture plates (Corning, Inc., Corning, NY, USA) and incubated using 2ml of Sphere Media (DMEM/F12 supplemented with 1:50 B27 (Invitrogen, Carlsbad, CA, USA), 20 ng/ml FGF (Invitrogen), 20 ng/ml EGF (Invitrogen), 4 mg/ml heparin (Sigma Aldrich, St. Louis, MO, USA) and 1% penicillin/streptomycin), as previously described [23]. For serial passages, spheres were collected every 5 days by centrifugation followed by enzymatic (trypsin treatment for 10 min at 37 °C) and single-cell suspension was counted and re-plated at the original density.

For adherent culture, 10^5^ tumor cells were seeded in 6-well culture plates (Corning) and grown in high glucose DMEM with 10% fetal bovine serum (Gibco, Waltham, MA, USA), 100μg/ml penicillin, and 100μg/ml streptomycin in a 5% CO_2_ incubator at 37°C.

**1.3. Collection of primary CRC tissue and single cell**

Briefly, fresh primary colorectal tumor specimens were cut into small fragments and then digested in DMEM/F12 medium containing 1.5mg/ml collagenase IV (Gibco), 20 ug/ml hyaluronidase (Sigma Aldrich), 1% penicillin/streptomycin at 37°C for 1 hour. The sample was filtered through 100μm-nylon filter and centrifuged for 5 min at 50 x g. Red blood cells were lysed using the red blood cell lysis buffer (Sigma Aldrich). Sing-cells were then washed twice, the final pellet were resuspended in PBS for further experiments.

**1.4. Hypoxia treatment**

In the first method, tumor cells were cultured in in DMEM with 1% FBS under physical hypoxic conditions (1% oxygen) for 3 days and then cultured under normoxic conditions (20% oxygen) for 3 days. The steps were repeated ten or more cycles. In the second method, tumor cells were cultured in DMEM with CoCl_2_ (300 μM) under normoxic conditions for 24 hours to harvest hypoxic tumor cells.

**1.5. Collection of normoxic and hypoxic cancer stem cells**

In the first method, tumor cells infected with the HRE-GFP lentivirus were cultured under normoxic or hypoxic conditions, respectively, and then stained with PE‑anti‑human CD133 at 4˚C for 15 min, then analyzed using a BD FACS Aria II flow cytometer. The population of CD133^+^GFP^-^ tumor cells were considered as normoxic cancer stem cells and the population of CD133^+^GFP^+^ tumor cells were considered as hypoxic cancer stem cells. In the second method, the ultra-low attachment plates (Corning) were used to enrich cancer stem cells. Tumor cells were cultured under normoxic or hypoxic conditions, respectively, and collected every 5 days.

**1.6. Collection of conditioned medium**

Before collection, cells were washed with PBS to eliminate all traces of serum in the cultures and incubated with fresh DMEM/F12 at 37°C, and 5% CO_2_ for 2 hours. CM was collected and centrifuged at 2000×g for 10 min at 4°C as previously described [19]. DNA and RNA hydrolase were added to the LCM and then boiled for 5 minutes to eliminate the effects of nucleic acids and extracellular proteins.

**1.7. Stable transfection of PGC-1a, LDHB and MCT1 shRNA**

Plasmids and lentivirus package were performed by GeneChem Company (Shanghai, China). Single cells were seeded with 3-5 × 10^4^ / ml in 6-well plates. The cells were infected with lentivirus for 24 hours at MOI of 50. Transfection efficiency was confirmed by reverse real-time PCR (RT-PCR) and Western blot analysis.

**1.8. Flow cytometry**

For the testing of normoxic CSCs from primary CRC tissues, the primary CRC cells were stained with APC-anti‐human EPCAM antibody and PE‐anti‐human CD133 antibody at 4 ℃ for 15 min, then stained with rabbit-anti-human HIF-1α at room temperature for 1h, followed by 488-conjugated-anti-rabbit secondary antibody.

For apoptosis, cell apoptosis was assayed using an Annexin V-7AAD Apoptosis Detection kit as previously described[50]. Briefly, 5 × 10^5^ tumor cells were collected and washed twice by cold PBS. Cells were centrifuged at 300 × g for 5 min at 4 °C. Then, cells were resuspended in 50 μL of 1× Binding Buffer and incubated with 2.5 μL of Annexin V (AV)-7AAD and 2.5 μL of PI staining Solution at RT for 10-15 min. 250 μL of 1× Binding Buffer was added into the mixture and flow cytometry was used to measure cell apoptosis.

**1.9. Invasion and wound healing assays**

For invasion assay, tumor cells were resuspended with 5 × 10^5^ /ml in serum-free DMEM medium or LCM and then pipetted in the upper chamber of transwell 24-well plates (8μm pores; Corning, NY, USA) at 200 ul per well. The membrane of upper chamber was coated with Matrigel (BD Biosciences). And 600 μl medium containing 10% FBS was added to the lower chamber. After incubation for 24 h, cells that did not invaded through the membrane were mechanically removed with a cotton swab. Next, 4% paraformaldehyde was used to fix the cells on the bottom surface of the membrane for 10 min, and then cells were stained with a 0.4% crystal violet solution. The invading cells were imaged using a digital microscopy (Nikon).

For wound healing assay, cells were seeded at 3×10^5^ cells/well in 6-well plates and exposed to treatments accordingly. After cells reached 100% confluence, a sterilized 200 μl pipette tip was used to make a straight scratch in the wells. Images were captured by a digital microscopy at 0h and 24h and the distances between one side of the scratch and the other at each time were measured. The distance of each scratch closure was closed gap.

**1.10. Tissue immunofluorescence**

Tissues were fixed in 4% paraformaldehyde then embedded in paraffin blocks and sectioned into 10 μm slices. Paraffin-embedded sections were treated with Instant Citrate Buffer Solution (RM-102C, LSI Medicine) or Target Retrieval Solution (S1699, Dako) appropriate to retrieve antigen. Then tissue sections were stained with the indicated antibodies, followed by visualization of Alexa-conjugated secondary antibodies or biotin-conjugated secondary antibodies followed by Streptavidin-HRP. Semi-quantitative analysis was performed with Image Pro Plus to identify the density (IOD/Area).

**1.11. Cell immunofluorescence**

Cells were grown on glass coverslips in 24-well plates at a density of 5 × 10^4^ cells/well for 24 h. Cells were washed with PBS and then fixed with 4% paraformaldehyde for 1 h at room temperature. Cells were permeabilized for 30 min in PBS supplemented with 0.4% Triton X-100 (Sigma-Aldrich) and 5% BSA (Sigma-Aldrich), and then labeled with the appropriate primary antibodies at 4 ℃ overnight and secondary antibodies for 1 h at room temperature.

**1.12. Real-time PCR**

To measure mitochondrial DNA(mtDNA) in CRC cell lines, total DNA was extracted from normoxic sphere-forming cells. The mitochondrial cytochrome C oxidase-1 (Co1) gene and the single-copy nuclear NADH dehydrogenase ubiquinone flavoprotein 1 (Ndufv1) genes were amplified by real-time PCR. Relative mitochondrial DNA content was calculated by assessing the relative levels of mtCo1 versus Ndufv1. Total RNA of cancer cells was isolated by using Trizol reagent (Takara) and 1μg of total RNA was reverse transcripted with PrimeScript RT Master Mix (Takara). Real-time PCR was performed by using TBGreen PCR master mix (Takara) and ABI PRISM 7300 Sequence Detection System (Applied Biosystem). All quantitative RT-PCR reactions were run in three independent experiments. Primers for the gene expression analysis are listed in Supplementary table 2.

**1.13. Western blot analysis**

Total protein of cancer cells was extracted by using RIPA buffer (50 mM Tris pH 7.4, 150 mM NaCl, 1 % Triton-X-100, 0.05 % sodium deoxycholate, 1 mM EDTA, 0.1 % SDS, protease inhibitor cocktail (Sigma) and PhosSTOP Phosphatase Inhibitor Cocktail (Roche)) and amount of protein was determined by BCA Protein Assay Kit (Thermo Fisher Scientific). After Electrophoresis and transfer, blots were incubated in 5% bovine serum albumin for 1 h and then incubated with primary antibodies at 4℃ overnight. Next day, blots were washed and incubated with secondary antibodies for 1 h and then detected by chemiluminescent substrate (Thermo Fisher Scientific) and imaging system (Fluor Chem FC2).

**1.14. Measurement of oxygen consumption rate**

Briefly, SW480 normoxic CSC cells were cultured with conditioned medium or control medium for 24 hours and seeded in culture plates at a density of 2 × 10^4^ cells per well. After being cultured at 37 °C with 5% CO2 overnight, the cells were washed twice with freshly prepared assay medium (10 mM glucose, 1 mM pyruvate, and 2 mM l-glutamine) and incubated in a low-CO_2_ incubator. Meanwhile, three compounds (1 µM oligomycin, 2 µM FCCP, and 0.5 µM antimycin A and rotenone) were added into specific reagent ports in the probe plates. Finally, calibration and measurement were executed with the Seahorse XFe24 Analyzer.

**1.15. ATP measurements**

ATP was measured using ATP assay kits (Life Technologies). After being diluted with dilution buffer, ATP detection reagent was added to a 96-well plate. After homogenization followed by centrifugation at 12,000 g at 4 °C for 5 minutes, the samples were added into the wells and mixed with the detection solution. A microplate luminometer was used to measure the luminescence intensity. ATP measurements were normalized to protein content.

**1.16. Cell counting kit-8 (CCK8) assay**

Cell proliferation was determined by CCK8 assays. Briefly, approximately 5000 cells were plated into each well of a 96-well plate and treated with corresponding processes. The cells were cultured for 24h. 10% of the CCK8 solution (5 mg/ml) (MedChemExpress) was added into each well for 3 h. The absorption of each well was then detected at 450 nm.

**1.17. *In vivo* mouse assays**

4 to 6-week-old female NOD/SCID mice were purchased from Beijing HFK Bioscience CO., LTD. (Beijing, China) and sorted randomly to experimental and control group (5 mice per group). SW480 normoxic CSC cells were resuspended at 10^7^/ml in PBS. Then mice were anesthetized with pentobarbital 60 mg/kg intraperitoneally, and 100μl of suspension were injected into the tail vein of each mouse. After 6-8 weeks, all mice were euthanized by CO_2_(3L/min) inhalation. All the lung metastasis sites were evaluated by histologic examination. Metastasis index is equal to total metastasis volume normalized by total lung volume.
