# Supplementary material for: Lactate promotes metastasis of normoxic colorectal cancer stem cells through PGC-1α-mediated oxidative phosphorylation
Source: Cell Death Dis. 2022 Jul 27;13(7):651. doi: 10.1038/s41419-022-05111-1 (PMC9329320; doi:10.1038/s41419-022-05111-1)
Supplement: Supplementary file 5 — Original western blots [file 41419_2022_5111_MOESM5_ESM.pdf]

Figure. 2h

ACTB

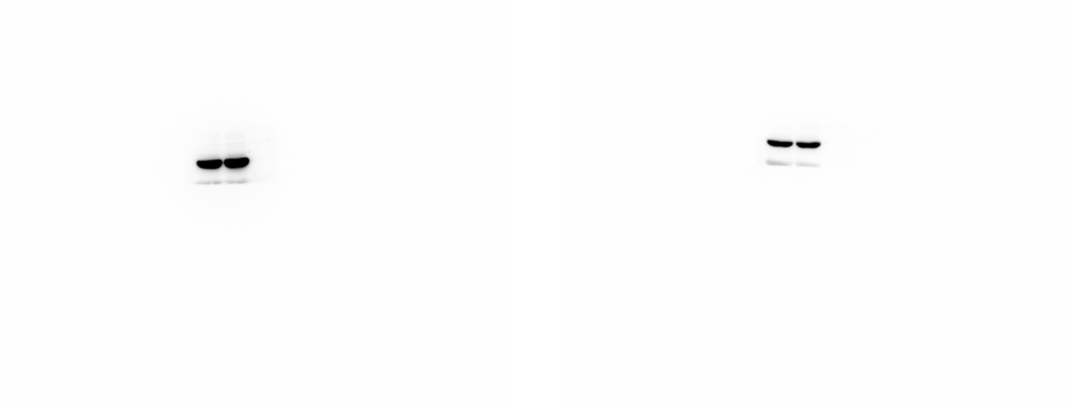

Vimentin

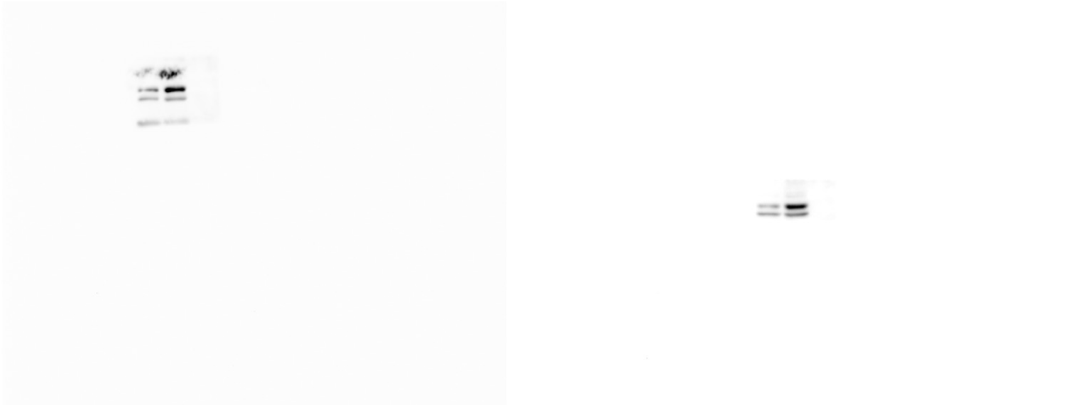

E-cadherin

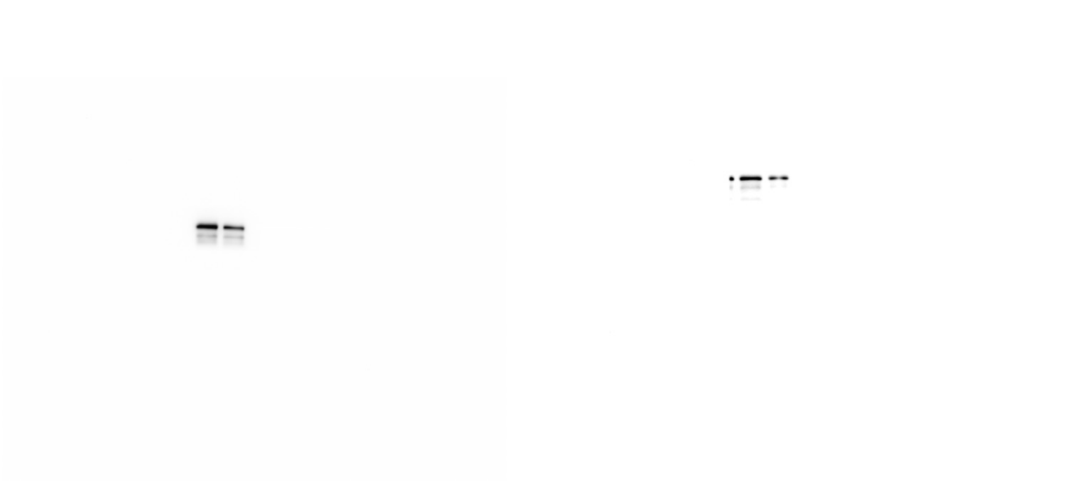

Figure. 3b

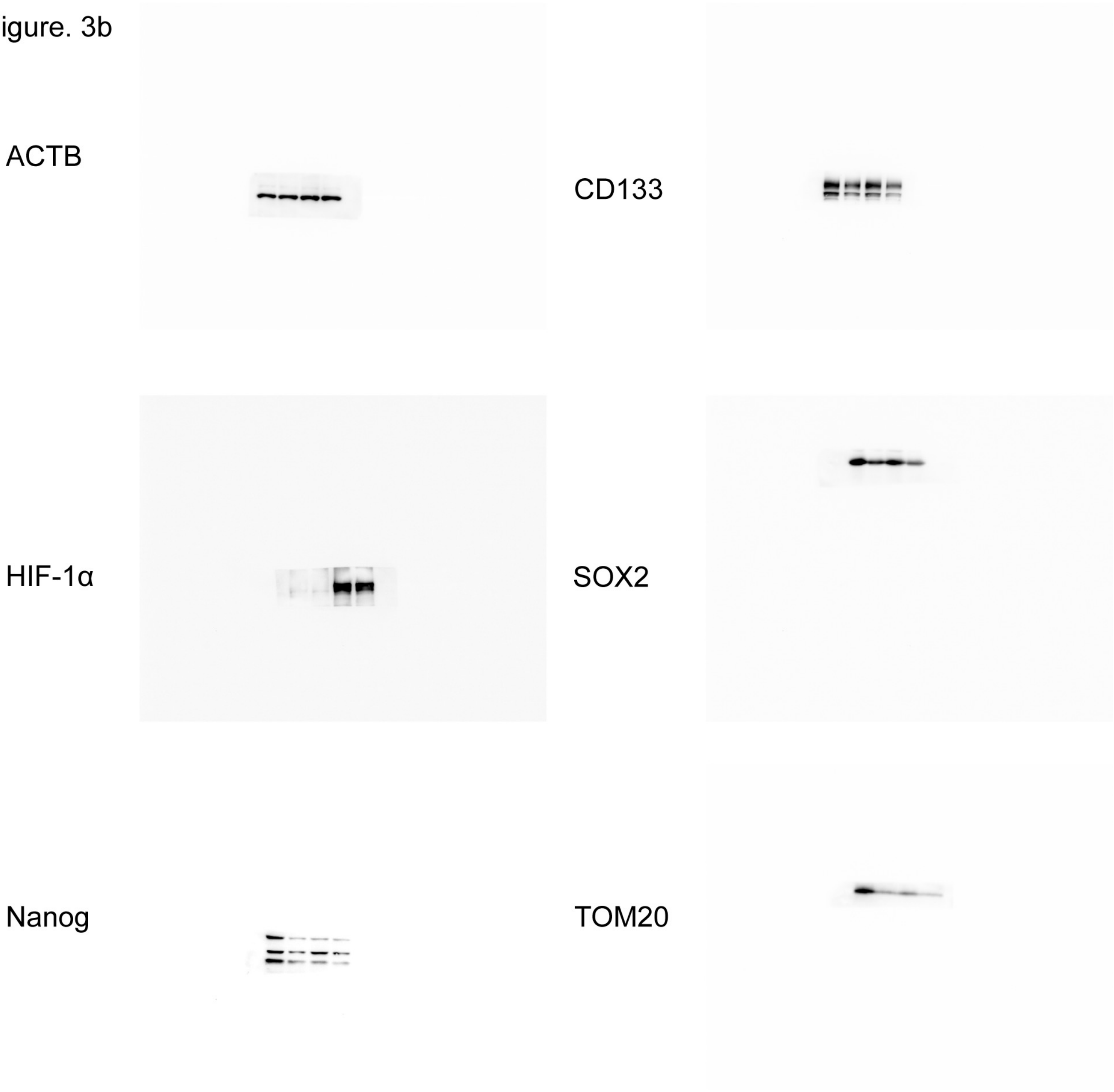

Figure. 3e

HIF-1α

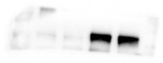

TOM20

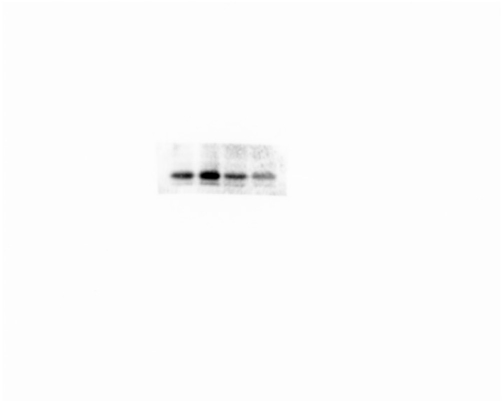

ACTB

Figure. 3f

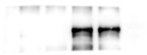

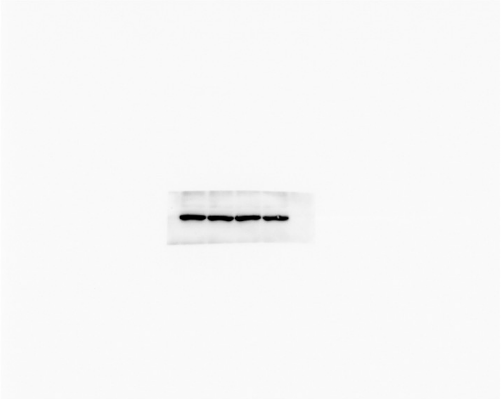

Figure. 3g

TOM20

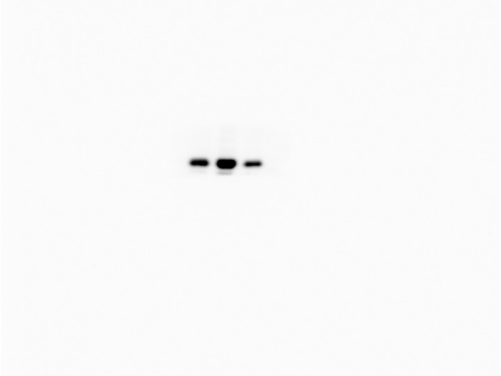

ACTB

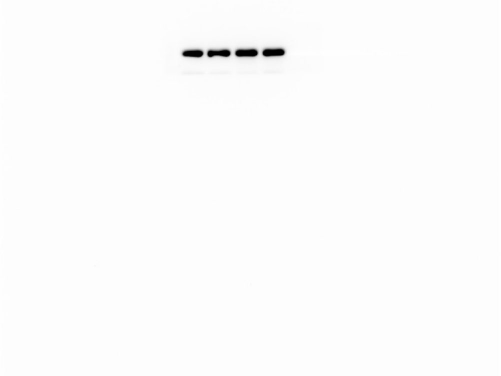

Figure. 4a

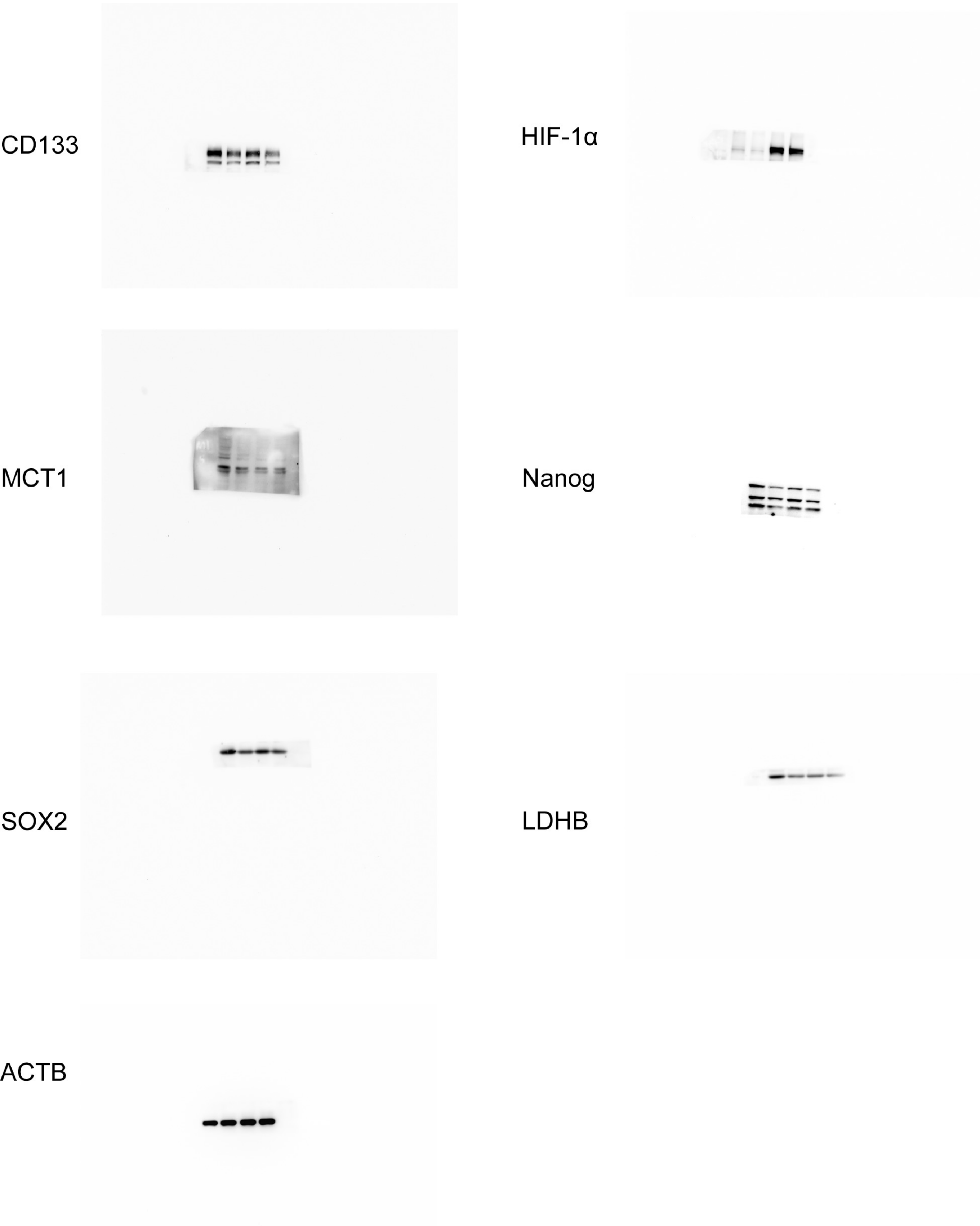

Figure. 4b

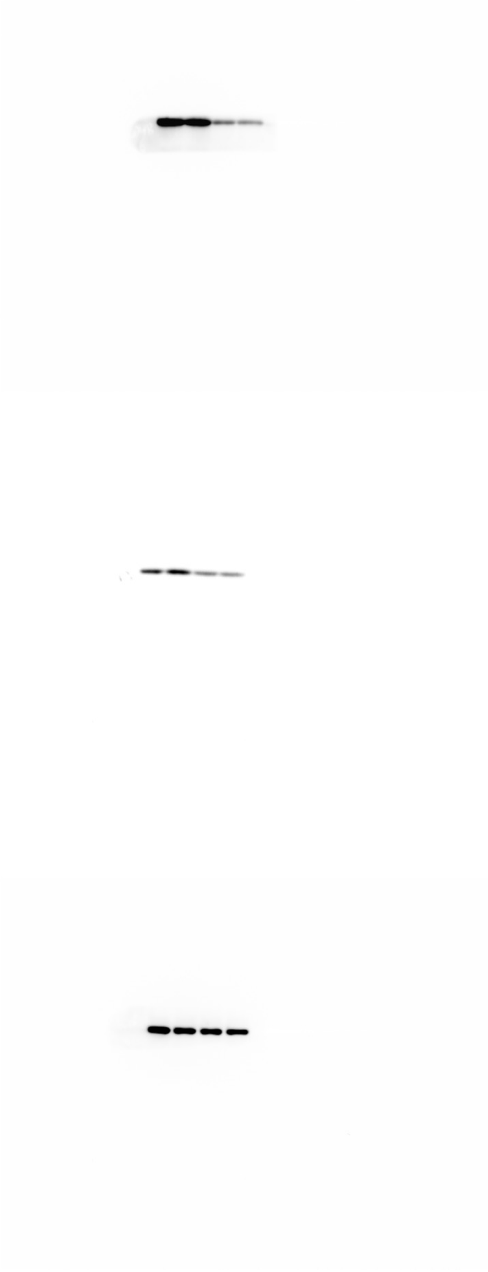

LDHB

TOM20

ACTB

Figure. 4c

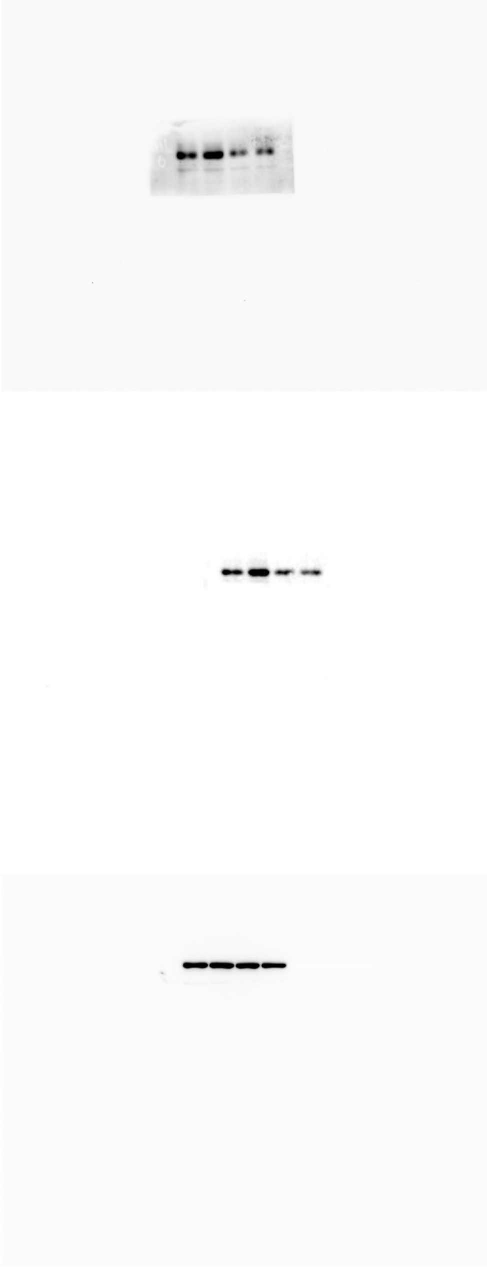

MCT1

TOM20

ACTB

Figure. 5a

CD133

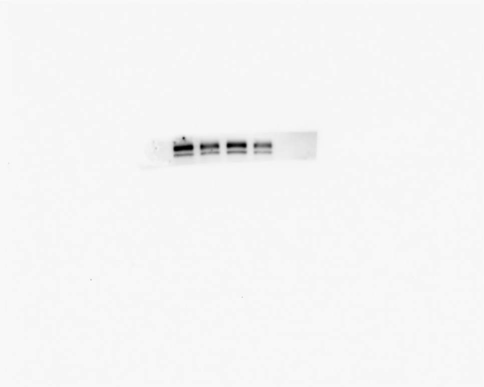

HIF-1α

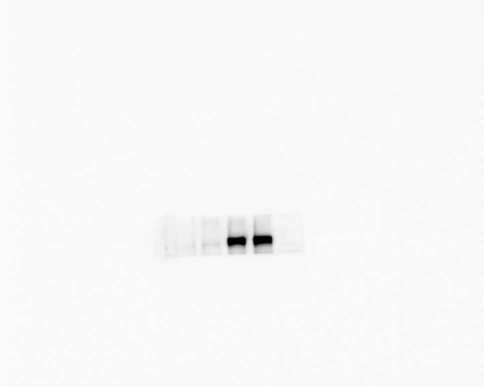

PGC-1α

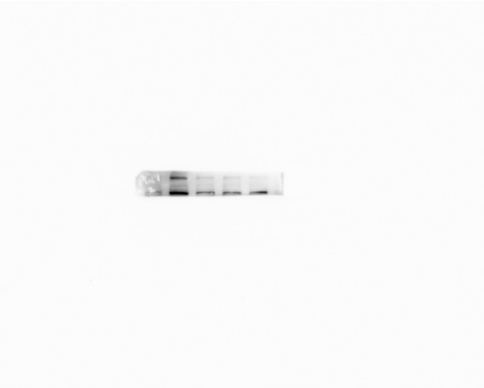

Nanog

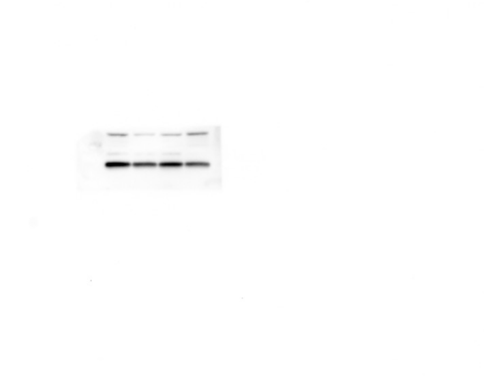

SOX2

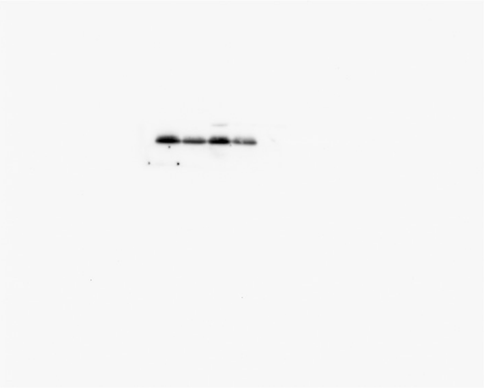

ACTB

Figure. 6a

HIF-1α

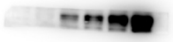

PGC-1α

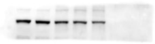

MCT1

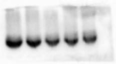

LDHB

TOM20

ACTB

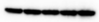

Figure.S2

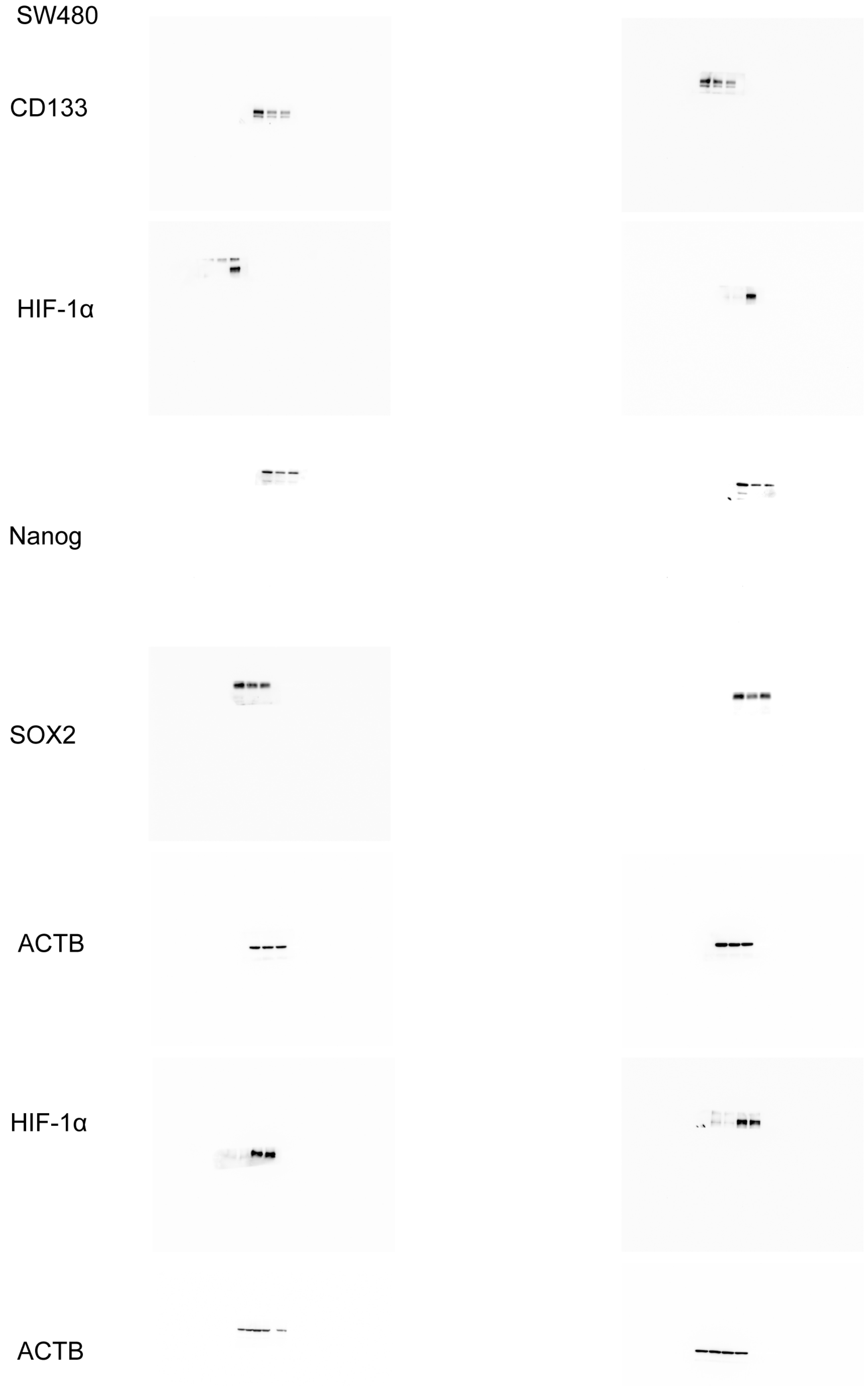

Figure.S2

LoVo

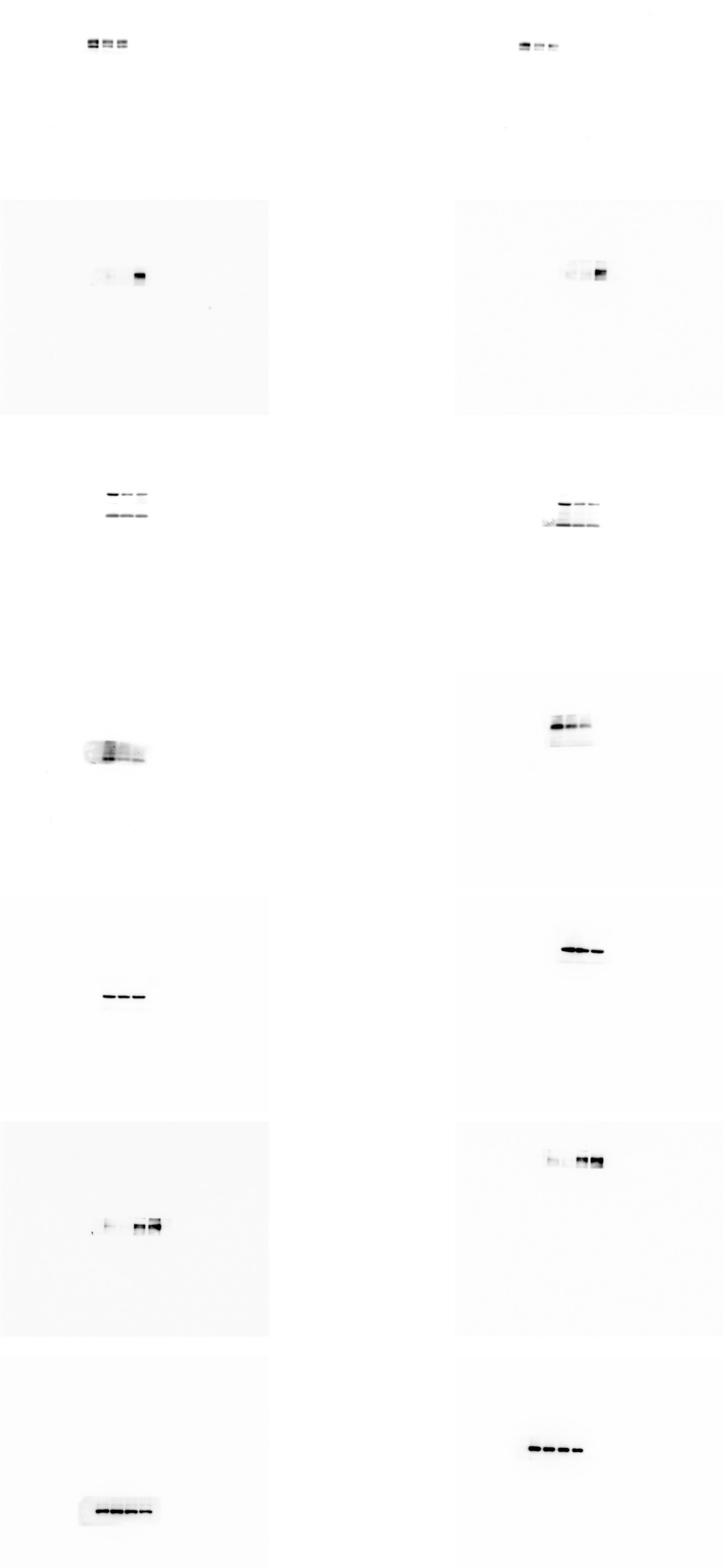

CD133

HIF-1α

Nanog

SOX2

ACTB

HIF-1α

ACTB

Figure.S2

XhCRC

CD133

HIF-1α

Nanog

SOX2

ACTB

HIF-1α

ACTB

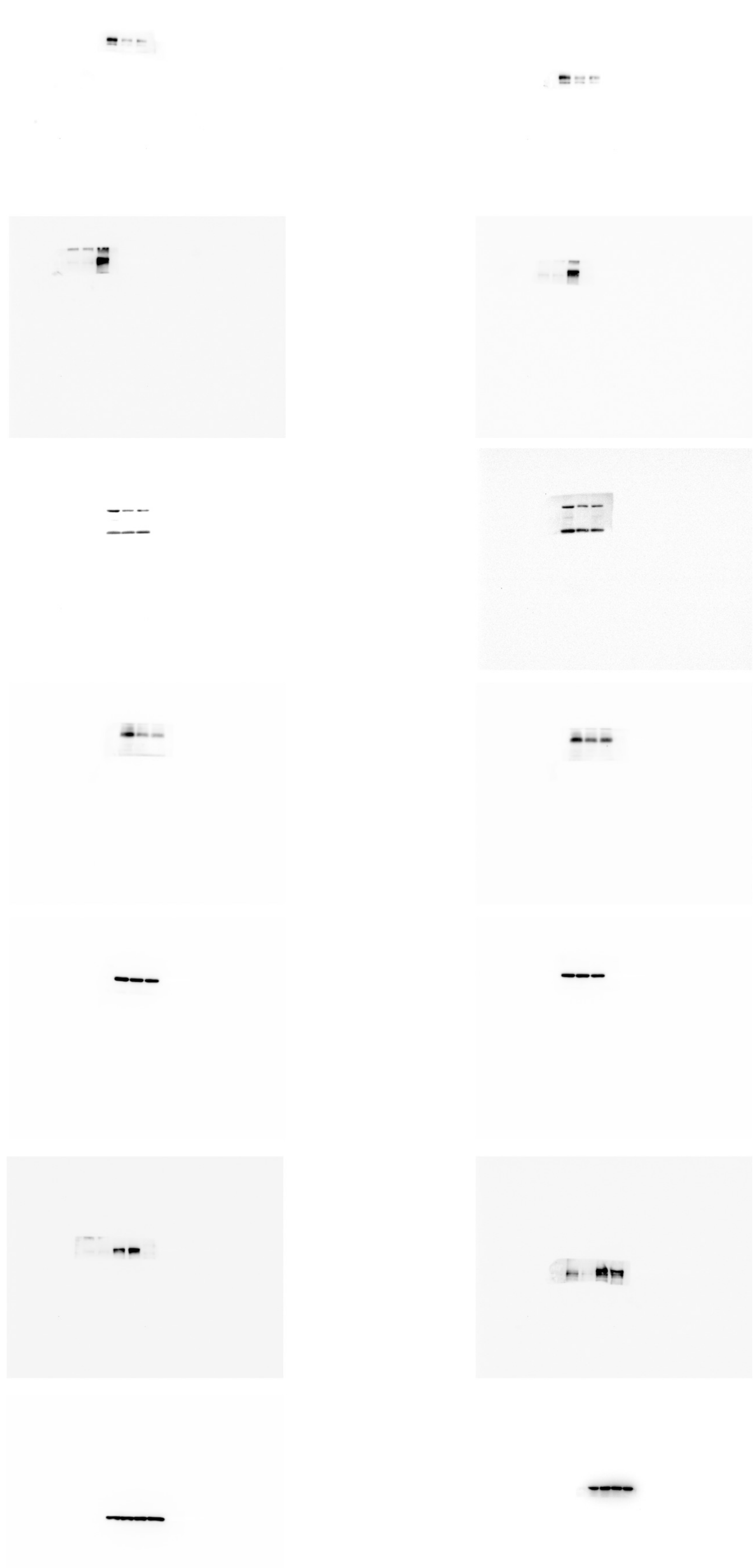

Figure. S3a

CD133

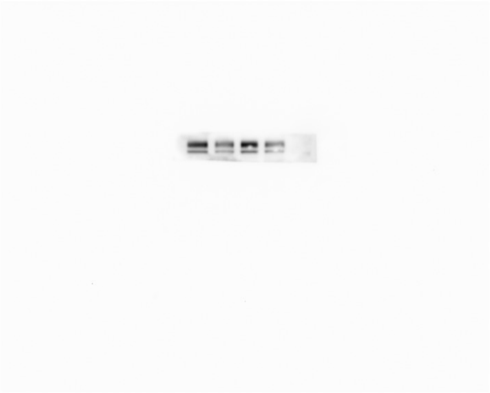

HIF-1α

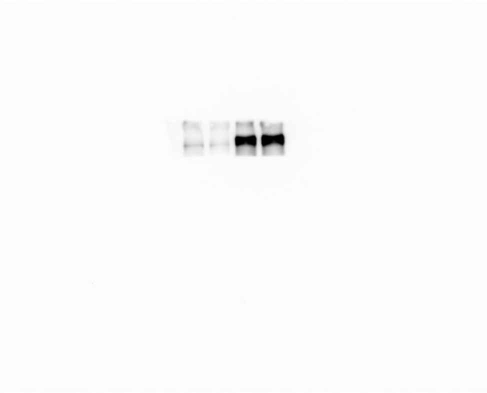

MCT1

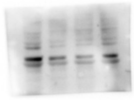

Nanog

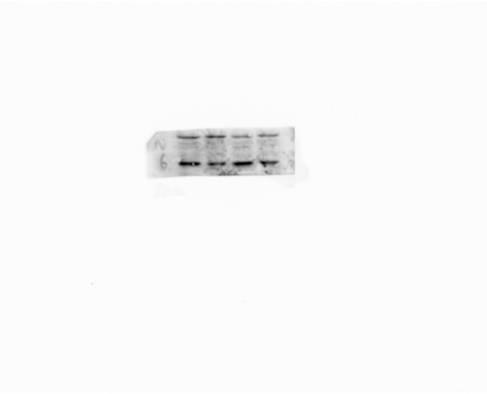

SOX2

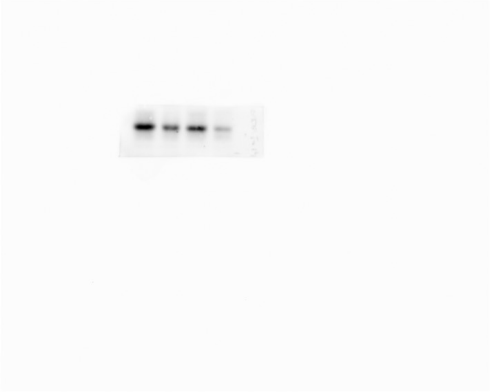

LDHB

ACTB
